# A comparative metabolomics analysis of the components of heartwood and sapwood in *Taxus chinensis* (Pilger) Rehd.

**DOI:** 10.1038/s41598-019-53839-2

**Published:** 2019-11-27

**Authors:** Fenjuan Shao, Lisha Zhang, Juan Guo, Xiaochun Liu, Wenhui Ma, Iain W. Wilson, Deyou Qiu

**Affiliations:** 1 State Key Laboratory of Tree Genetics and Breeding, Key Laboratory of Tree Breeding and Cultivation of National Forestry and Grassland Administration, Research Institute of Forestry, Chinese Academy of Forestry, Beijing, 100091 China; 2 Research Institute of Wood Industry, Chinese Academy of Forestry, Beijing, 100091 China; 3 Gansu Forestry and Grassland Administration, Lanzhou, 730030 China; 4 Liangdang Forestry Administration, Gansu Province, 742400 China; 5 CSIRO Agriculture and Food, PO Box 1700, Canberra, ACT 2601 Australia

**Keywords:** Bioinorganic chemistry, Metabolomics

## Abstract

*Taxus chinensis* is a well-known gymnosperm with great ornamental and medicinal value. Its purple red brown heartwood (HW) has many attributes such as straight texture, high density, mechanical strength, rich elasticity and corrosion resistance that is highly prized commercially. *T. chinensis* sapwood (SW), in comparison, lacks these important traits. At present, little is known about the differences of metabolites between the SW and HW in *T. chinensis*. Widely targeted metabolic profiling was performed to analyze the metabolic profiles of HW and SW in *T. chinensis* using Liquid Chromatography-Electrospray Ionization-Mass Spectrometry (LC-EI-MS). A total of 607 metabolites were detected in HW and SW. Among them, 146 metabolites were significantly higher, and 167 metabolites significantly lower, in HW as compared to SW. These differential metabolites were mainly involved in metabolic pathways and biosynthesis of secondary metabolites, such as flavonoids, flavone and flavonol, phenylpropanoids and antibiotics. Moreover, 71 flavonoids and isoflavones were found to be significantly different between HW and SW. Our results show the difference of components between the HW and SW, which has potential significance to further elucidate the mechanism of HW color formation. The results will provide insight into the metabolites associated with wood color formation and useful information for understanding the metabolites associated with wood quality.

## Introduction

Wood, the secondary xylem of trees, is one of the most important source of materials and energy in the world^1–3^. It is important to human life, for example, wood can be as fuel for cooking as well as raw material for buildings^2,3^. In addition, it is also important to industrial, energy and environmental fields, such as renewable feedstock for pulp, biofuels, biomass energy and carbon sink^2–4^. Many tree species can be divided into sapwood (SW) and heartwood (HW) (Celedon and Bohlmann, 2018). HW is usually the dead inner wood, and reserve materials are converted into HW substances, while SW is the living, outermost portion wood that contains reserve materials^5^. Chemical composition plays an important role in wood properties, affecting physical and mechanical properties, natural durability, color and utilization of wood^6,7^. Compared to the SW, HW has an important economic value due to the natural decay resistance, wood color, wood fragrance and pharmaceutical substances^8^.

Wood color is an important trait for wood quality in the forest industry. It plays an important role in the processing of wood products, such as furniture, wood carving and building industries etc. It has been shown that the formation of wood color is due to the existence of secondary metabolites in the HW, including stilbenes, flavonoids and other phenolic compounds^5^, but the components of these secondary metabolites have not been investigated, and how these secondary metabolites affect wood color formation remain largely unknown. Predominately, research in wood color has mainly been focused on functional improvement through chemical methods^9–11^. The molecular genetic mechanisms of wood color formation are poorly understood. If the mechanism of wood color formation is understood, it may be possible to control wood color in trees via molecular genetics.

*Taxus chinensis*, belongs to the Taxus family, also known as yew, and is a well-known gymnosperm with great ornamental and medicinal value^12^. The bark of *T. chinensis* can produce paclitaxel, which has been widely used in the treatment of lung, ovarian and breast cancer^13–15^. In addition, the wood of Taxus has high commercial value because of its good aesthetic appearance, straight texture, high density, mechanical strength, rich elasticity, corrosion resistance, white yellowish SW and purple red brown colored HW. However, little is known about the metabolites variation between the SW and HW in *T. chinensis*.

Metabolomics is a valuable approach for the high-throughput and comprehensive study of complex metabolic compositions, and has been widely applied in plants^16–20^. Mass spectrometry methods have been used for detection and quantification of metabolites^16–18^. With the aim to investigate the metabolites variation between the SW and HW in *T. chinensis*, widely targeted metabolomics approach using liquid chromatography tandem mass spectrometry (LC-MS/MS) was performed. The metabolites in SW and HW were identified, the differences in metabolite profile in SW and HW compared. Our results are potentially useful for the further elucidation of the mechanism of HW color formation. The results will provide insight into the molecular mechanisms of wood formation and useful information for improved wood quality.

## Results and Discussion

### Metabolic profiling of heartwood and sapwood based on LC-MS/MS

**Figure 1:**
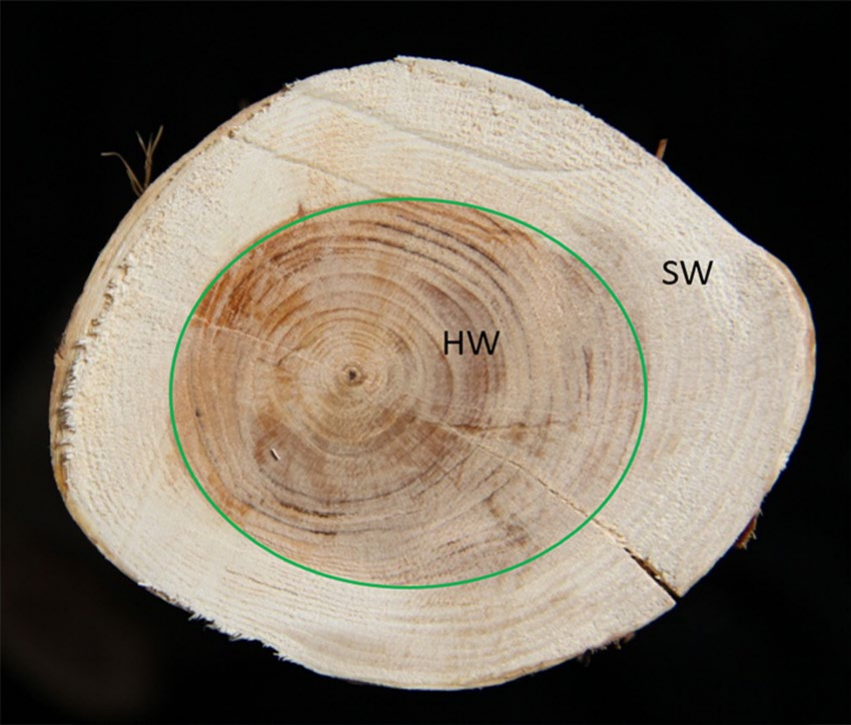
The heartwood and sapwood in *T. chinensis* (Pilger) Rehd. (HW: heartwood, SW: sapwood).

SW and HW were collected from the branch from an approximately 30-year-old of *T. chinensis* (Pilger) Rehd. The outer wood tissue with a pale yellow color is defined as SW and the central tissue with a red or dark-brown color is characterized as HW (Fig. 1). In order to investigate the components of HW and SW in *T. chinensis*, widely targeted metabolic profiling was performed to analyze the metabolic profiles of HW and SW in *T. chinensis* by using the Liquid Chromatography-Electrospray Ionization-Mass Spectrometry. Metabolomics data of HW and SW were processed using System Software Analyst (Version 1.6.3). Metabolites were quantitatively analyzed following collection of secondary data using the MRM model, as a result, a total of 607 metabolites were identified in HW and SW, including 52 lipids, 98 organic acids and their derivatives, 43 nucleotides and their derivatives, 132 flavonoids, 55 amino acids and their derivatives, 36 alkaloids, 93 phenylpropanoids, 12 vitamins, 17 terpenes, 21 carbohydrates and 48 others (supplementary file 1). These metabolites are involved in the most of primary and secondary metabolisms. Among these metabolites, the most abundant metabolites are the flavonoids, suggesting the flavonoids play a role in the process of the wood color formation.

### Principal component analysis (PCA) and cluster analysis of HW and SW

**Figure 2:**
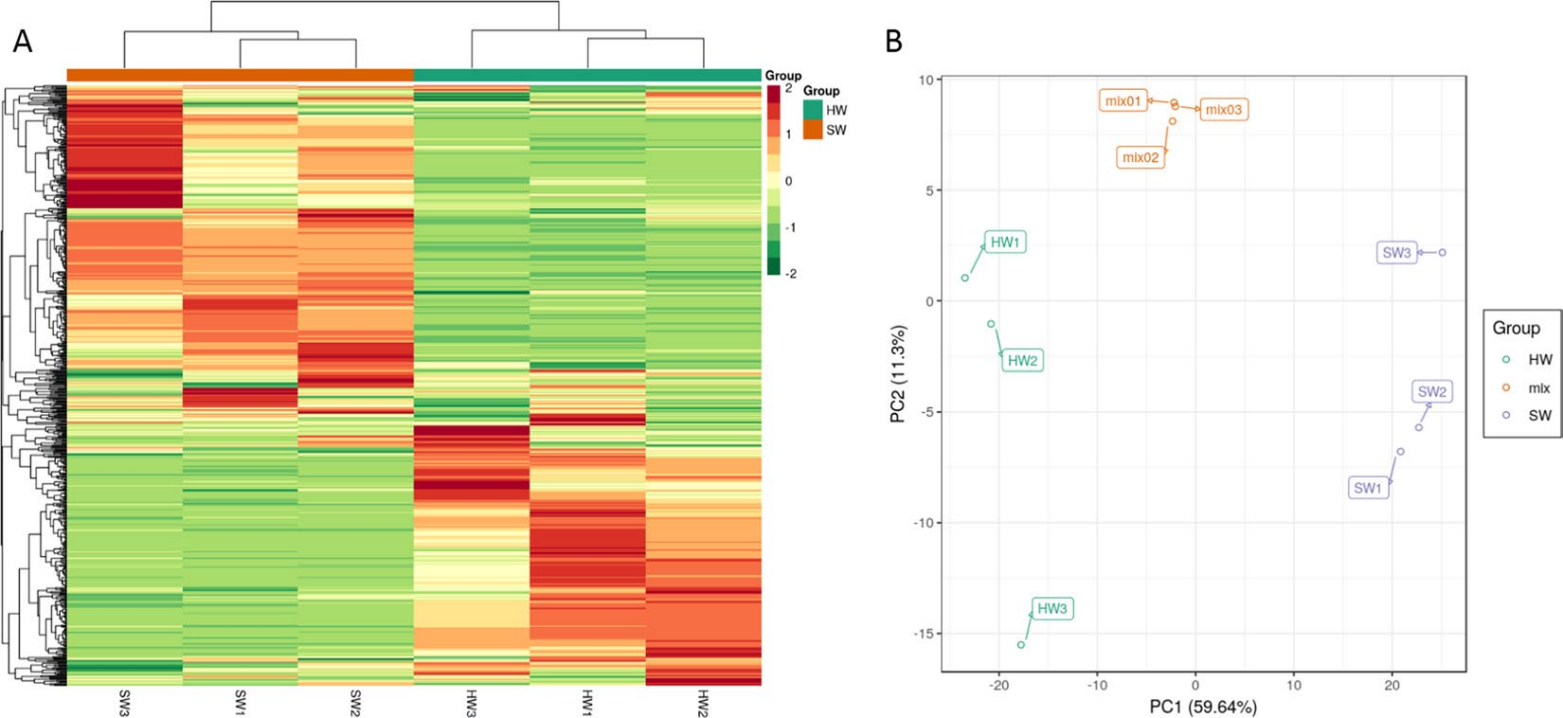
(**A**) Heatmap of the metabolites in heartwood and sapwood; (**B**) PCA score plot of the metabolites in the heartwood and sapwood.

In order to investigate the HW and SW metabolic differences and multivariate, mixed samples of the HW and SW were prepared and performed to metabolic profiling using the Liquid Chromatography-Electrospray Ionization-Mass Spectrometry. Principal component analysis (PCA) is used to reveal the differences in metabolic profiles between the HW and SW. In the PCA plot (Fig. 2A), the mixed samples grouped together, indicating that the mixed samples had similar metabolic profiles. PCA clearly grouped these samples into distinct clusters, indicating significant differences in metabolites between the HW and SW. Hierarchical cluster analysis (HCA) of the metabolites of the HW and SW was performed. The results of all detected metabolites are shown in a heatmap (Fig. 2B), which indicates the significant differences in the relative abundance of metabolites between HW and SW. The metabolite profile of the HW and SW are clearly divided into two main clusters based on the differences in accumulation patterns on the heatmap, suggesting a clear variation in terms of the metabolites abundance in the HW and SW.

### Differential metabolite analysis based on partial least squares-discriminant analysis (PLS-DA)

**Figure 3:**
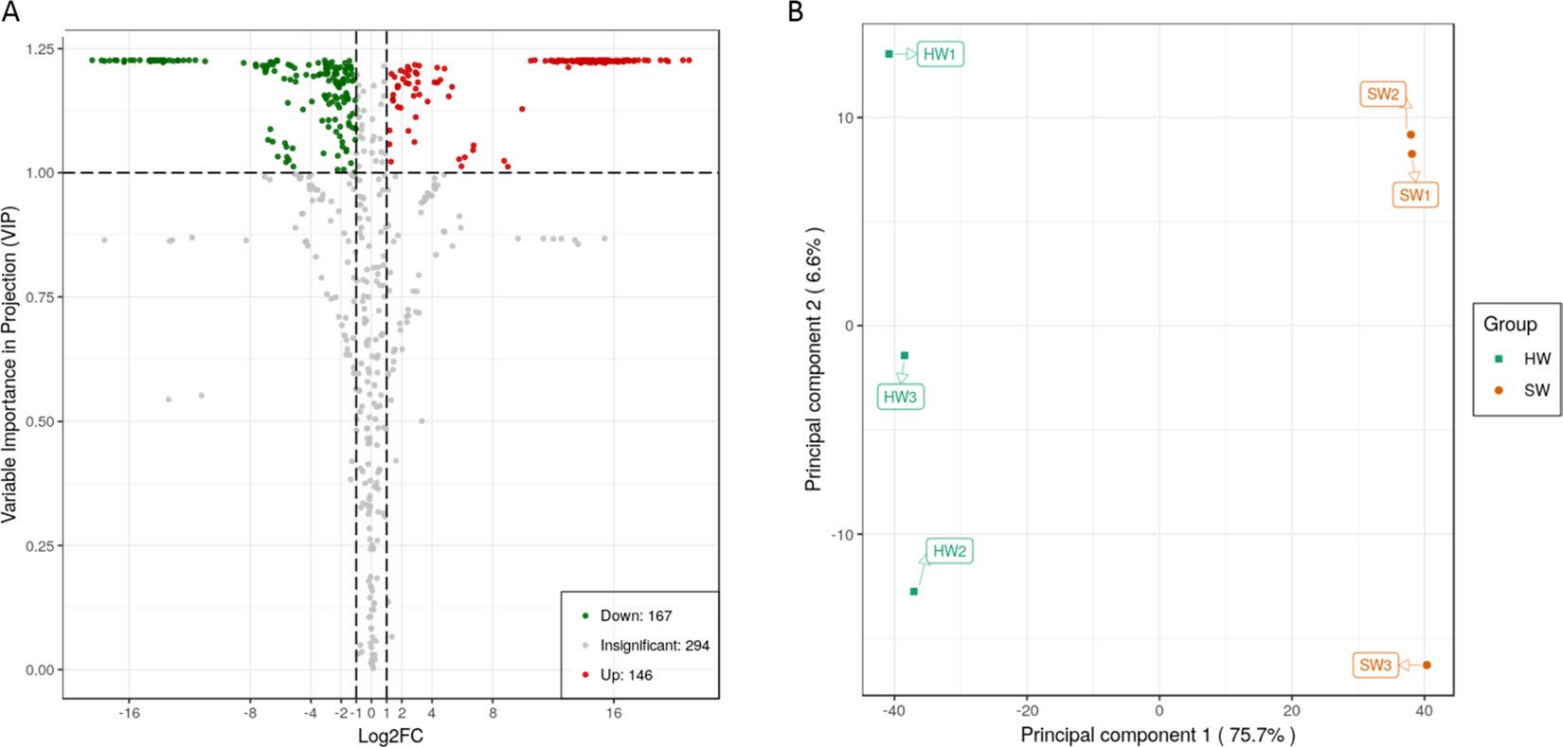
(**A**) The volcano plot of the differential metabolites in the heartwood and sapwood. Green dots represent down-regulated metabolites, red spots represent up-regulated metabolites and gray represent insignificant difference metabolites. (**B**) PLS-DA model plot of the differential metabolites in the heartwood and sapwood.

To obtain the metabolic differences between the HW and SW, the PLS-DA models was used to screen differential compounds between two groups of samples (Fig. 3). Moreover, a fold-change score ≥2 or ≤0.5 among the metabolites with a VIP value >1 was used to identify differential metabolites. The screening results have been illustrated using Volcano plots (Fig. 3). A total of 313 significant differences of metabolites were identified, of these differential metabolites there were 146 metabolites significantly higher and 167 metabolites significantly lower between the HW and SW (supplementary file 2). The top 30 metabolites that were higher in HW are shown in Table 1, and among them, 11 flavonoids were found to be significantly different between the HW and SW. These metabolites may be considered to be the representative differential metabolites for the HW and SW, influencing their wood properties especially the wood color.

**Table 1:** The top 30 significant differences of metabolites in heartwood and sapwood.

Number	Compounds	Class	VIP	Fold_Change	Log2FC
1	C-pentosyl-apigenin O-p-coumaroylhexoside	Flavone	1.227	2051851.852	20.968
2	β-Caryophyllene	Terpene	1.227	1548148.148	20.562
3	Dehydrocorydaline	Alkaloids	1.224	786666.667	19.585
4	Ayanin	Flavonol	1.227	708148.148	19.434
5	Xanthotoxol	Phenylpropanoids	1.227	555925.926	19.085
6	Vitamin A	Vitamins and derivatives	1.226	314444.444	18.262
7	Ethyl 3,4-Dihydroxybenzoate	Organic acids and derivatives	1.227	266296.296	18.023
8	Schizandrin B	Phenylpropanoids	1.227	200740.741	17.615
9	N-Methyltryptamine	Alkaloids	1.227	165925.926	17.340
10	Ellagic acid	Polyphenol	1.225	162481.481	17.310
11	O-methylChrysoeriol 8-C-hexoside	Flavone	1.226	131111.111	17.000
12	Malvidin 3-O-glucoside (Oenin)	Anthocyanins	1.226	130888.889	16.998
13	Crocetin	Terpene	1.226	119666.667	16.869
14	Chrysin O-hexoside	Flavone	1.226	119259.259	16.864
15	16-Hydroxy hexadecanoic acid	Lipids	1.227	115148.148	16.813
16	Malvidin 3-O-galactoside	Anthocyanins	1.225	101592.593	16.632
17	4-Hydroxy-3,5-diisopropylbenzaldehyde	Organic acids and derivatives	1.226	100814.815	16.621
18	Prehelminthosporolactone	Others	1.224	100555.556	16.618
19	Ethyl cinnamate	Phenylpropanoids	1.226	81074.074	16.307
20	Luteolin O-hexosyl-O-pentoside	Flavone	1.223	80074.074	16.289
21	O-methylChrysoeriol 7-O-hexoside	Flavone	1.226	75259.259	16.200
22	O-methylChrysoeriol 5-O-hexoside	Flavone	1.224	69925.926	16.094
23	LysoPC 16:2 (2n isomer)	Lipids	1.225	68037.037	16.054
24	1,10-decanediol	Alcohols	1.224	59000.000	15.848
25	8-Methoxypsoralen	Phenylpropanoids	1.226	54333.333	15.730
26	Psoralen	Phenylpropanoids	1.224	51148.148	15.642
27	Myricetin	Flavonol	1.226	50074.074	15.612
28	Glabridin	Flavonoid	1.227	48370.370	15.562
29	Camptothecin	Alkaloids	1.227	47222.222	15.527
30	Phytocassane C	Terpene	1.226	46925.926	15.518

### Metabolic pathway analysis of differential metabolites

**Figure 4:**
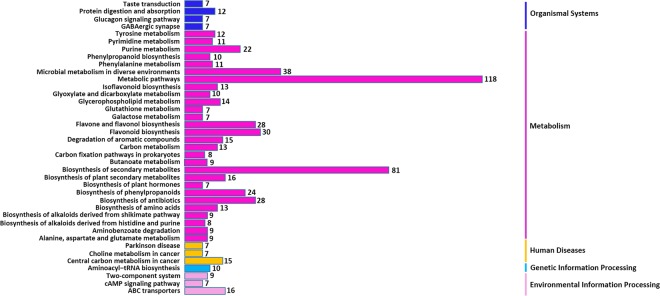
The distribution of metabolic pathways of the differential metabolites.

To obtain the pathway information of differential metabolites, the differential metabolites between the HW and SW were mapped to the Kyoto Encyclopedia of Genes and Genomes (KEGG) database (http://www.genome.jp/kegg/). The results are shown in Fig. 4, these differential metabolites are mainly involved in metabolic pathways and biosynthesis of secondary metabolites, such as flavonoids, flavone and flavonol, phenylpropanoids and antibiotics etc. Flavonoids, flavone and flavonol were included in phenylpropanoids, which are a large class of plant secondary metabolites derived from phenylalanine in plants. In addition to flavonoids, it also includes monolignols, phenolic acids, stilbenes, and coumarins^21–23^. As we know, cellulose, hemicellulose and lignin are the major components of wood, but they do not exhibit color. It has been shown that the wood color is due to the existence of colored extractives contained in the wood. These colored extractives turn into dark color from a pale color by oxidation, polymerization and polymerization with wood main components over time^24^. Previous studies have shown that the extractives of some important colored woods were the flavonoids^25,26^, suggesting that the flavonoid metabolites identified in this study may explain the difference in wood color between the HW and SW in *T. chinensis*.

### Flavonoids identified in HW

Wood color is one of the important properties related to the wood utilization^24–26^. Flavonoids as the major pigment molecules are widely studied in plants and it has been shown that the flavonoids play a crucial role in the formation of wood color^24–26^. So the flavonoids in HW were selected for further analysis. The results are shown in Table 2, among 313 differential metabolites, we identified a total of 71 flavonoids and isoflavones, including 28 flavones, 3 anthocyanins, 16 flavonols, 11 flavonoids, 8 flavanones and 5 isoflavones. 42 flavonoids were higher and 29 flavonoids lower in HW compared to SW. Among them, the top 5 metabolites that were higher in HW were C-pentosyl-apigenin O-p-coumaroylhexoside, Ayanin, O-methylChrysoeriol 8-C-hexoside, malvidin 3-O-glucoside and chrysin O-hexoside, but the top 5 metabolites significantrly lower in HW compared to SW were 2′-hydroxydaidzein, 8-C-hexosyl chrysoeriol O-hexoside, cyanidin 3-O-malonylhexoside, isorhamnetin 5-O-hexoside and isorhamnetin 3-O-glucoside. These differential flavonoids metabolites show the difference of components between the HW and SW in *T. chinensis*, but the mechanism for the difference of components between the HW and SW is currently unknown and needs to be further investigated. Moreover, how these differential metabolites affect the wood color of HW also need to be further investigated.

**Table 2:** A list of flavonoid metabolites identified in heartwood and sapwood.

Compounds	Class	VIP	Fold_Change	Log2FC	Regulate
Cyanidin 3-O-malonylhexoside	Anthocyanins	1.227	0.00	−14.63	down
O-methylChrysoeriol 5-O-hexoside	Flavone	1.224	69925.93	16.09	up
Chrysoeriol 5-O-hexoside	Flavone	1.226	0.00	−14.29	down
Chrysin O-hexoside	Flavone	1.226	119259.26	16.86	up
O-methylnaringenin C-pentoside	Flavone	1.207	5.66	2.50	up
O-methylChrysoeriol 8-C-hexoside	Flavone	1.226	131111.11	17.00	up
Ayanin	Flavonol	1.227	708148.15	19.43	up
O-methylChrysoeriol 7-O-hexoside	Flavone	1.226	75259.26	16.20	up
Isorhamnetin O-hexoside	Flavonol	1.226	0.00	−14.31	down
Selgin O-hexosyl-O-hexoside	Flavone	1.226	25481.48	14.64	up
Luteolin O-hexosyl-O-pentoside	Flavone	1.223	80074.07	16.29	up
Apigenin O-hexosyl-O-pentoside	Flavone	1.223	4907.41	12.26	up
Chrysin 5-O-glucoside (Toringin)	Flavone	1.031	71.33	6.16	up
Luteolin 3′,7-di-O-glucoside	Flavone	1.226	0.00	−12.42	down
Isorhamnetin 5-O-hexoside	Flavonol	1.227	0.00	−14.51	down
Chrysoeriol 7-O-hexoside	Flavone	1.227	0.00	−14.34	down
C-hexosyl-isorhamnetin O-hexoside	Flavone	1.227	32814.81	15.00	up
6-C-hexosyl chrysoeriol O-hexoside	Flavone	1.227	38962.96	15.25	up
C-pentosyl apigenin O-salicyloyl hexoside	Flavone	1.223	38629.63	15.24	up
C-pentosyl-apigenin O-p-coumaroylhexoside	Flavone	1.227	2051851.85	20.97	up
8-C-hexosyl chrysoeriol O-hexoside	Flavone	1.226	0.00	−15.69	down
Tricin 5-O-β-guaiacylglycerol	Flavone	1.173	39.99	5.32	up
Tricin O-rhamnoside	Flavone	1.225	12848.15	13.65	up
Apigenin C-hexosyl-O-rutinoside	Flavone	1.057	2.27	1.19	up
Tricin 7-O-hexoside	Flavone	1.225	36296.30	15.15	up
Tricin 4′-O-β-guaiacylglycerol	Flavone	1.212	8200.00	13.00	up
Tricin O-eudesmic acid	Flavone	1.225	21555.56	14.40	up
Di-O-methylquercetin	Flavonol	1.157	0.32	−1.63	down
Kaempferol 7-O-rhamnoside	Flavonol	1.210	0.21	−2.25	down
Chrysoeriol	Flavone	1.095	0.36	−1.47	down
Kaempferol 3-O-rutinoside (Nicotiflorin)	Flavonol	1.154	0.19	−2.40	down
Naringenin	Flavanone	1.182	17.86	4.16	up
Apigenin	Flavone	1.202	0.30	−1.72	down
Malvidin 3-O-galactoside	Anthocyanins	1.225	101592.59	16.63	up
Malvidin 3-O-glucoside (Oenin)	Anthocyanins	1.226	130888.89	17.00	up
Xanthohumol	Flavanone	1.225	7825.93	12.93	up
Myricetin	Flavonol	1.226	50074.07	15.61	up
Isorhamnetin 3-O-neohesperidoside	Flavonol	1.156	0.14	−2.80	down
Isorhamnetin	Flavonol	1.144	13.00	3.70	up
7-O-Methyleriodictyol	Flavanone	1.073	0.23	−2.09	down
Kaempferol 3-O-robinobioside (Biorobin)	Flavonol	1.182	0.19	−2.38	down
Isohemiphloin	Flavone	1.226	3637.04	11.83	up
Hesperetin 7-rutinoside (Hesperidin)	Flavanone	1.093	0.24	−2.07	down
Naringenin chalcone	Flavanone	1.181	20.49	4.36	up
Aromadedrin (Dihydrokaempferol)	Flavonol	1.225	35185.19	15.10	up
3-Hydroxyflavone	Flavonol	1.225	36000.00	15.14	up
Liquiritigenin	Flavanone	1.225	0.00	−10.98	down
Biochanin A	Isoflavone	1.062	0.26	−1.92	down
2′-Hydroxydaidzein	Isoflavone	1.227	0.00	−15.93	down
2′-Hydroxygenistein	Isoflavone	1.226	45962.96	15.49	up
Afzelechin (3,5,7,4′-Tetrahydroxyflavan)	Flavanone	1.013	0.03	−5.15	down
3,7-Di-O-methylquercetin	Flavonol	1.062	7.16	2.84	up
Prunetin	Isoflavone	1.043	0.32	−1.66	down
Tricetin	Flavone	1.150	0.20	−2.29	down
Rhamnetin (7-O-methxyl quercetin)	Flavonol	1.227	16259.26	13.99	up
Fustin	Flavonol	1.200	0.19	−2.43	down
Rotenone	Isoflavone	1.226	35777.78	15.13	up
Homoeriodictyol	Flavanone	1.150	0.34	−1.55	down
4,2′,4′,6′-Tetrahydroxychalcone	Flavone	1.187	23.28	4.54	up
Narcissoside	Flavonoid	1.106	0.14	−2.83	down
Orientin	Flavonoid	1.226	23074.07	14.49	up
5,7-Dihydroxychromone	Flavonoid	1.088	0.49	−1.03	down
Herbacetin	Flavonoid	1.172	0.23	−2.13	down
Isorhamnetin 3-O-glucoside	Flavonoid	1.227	0.00	−14.36	down
Farrerol	Flavonoid	1.092	0.14	−2.84	down
Puararin	Flavonoid	1.225	4048.15	11.98	up
Diosmin	Flavonoid	1.225	2848.15	11.48	up
Tectochrysin	Flavonoid	1.226	44629.63	15.45	up
Tectorigenin	Flavonoid	1.226	26111.11	14.67	up
Glabridin	Flavonoid	1.227	48370.37	15.56	up
Chalcone	Flavonoid	1.224	10122.22	13.31	up

## Materials and Methods

### Plant materials

The wood samples were collected from the branch of an approximately 30-year-old T. chinensis (Pilger) Rehd. in May 2015 from Liangdang County (106°25′E, 33°41′N) in Shanxi province of China. The HW and SW was separated on the basis of color, the inner wood was HW and the outer SW. Three independent biological replicates were tested for each sample. Samples were dried naturally and ground to a fine powder for usage.

### Sample preparation for metabolite profiling

The HW and SW was separated by Tungsten steel knife. The dried HW and SW samples were ground using a mixer mill (MM 400, Retsch). The powder about 100 mg was extracted with 1.0 mL 70% methanol overnight at 4 °C, then the extraction was centrifuged at 10,000 g for 10 min. The extracts were absorbed and filtrated before LC-MS analysis. The mixed samples of the HW and SW as a quality control were prepared according to the above methods.

### Liquid chromatographic mass spectrometry

LC-ESI-MS/MS system (HPLC, Shim-pack UFLC SHIMADZU CBM30A system, MS, Applied Biosystems 6500 Q TRAP) were used to analyze the sample extracts. HPLC analyses used Waters ACQUITY UPLC HSS T3 C18 (1.8μm, 2.1 mm*100 mm) column.

solvent system of mobile phase was 0.04% acetic acid in water and 0.04% acetic acid in acetonitrile; the gradient program was as follows: 95:5 V/V at 0 min, 5:95 V/V at 11.0 min, 5:95 V/V at 12.0 min, 95:5 V/V at 12.1 min, 95:5 V/V at 15.0 min; the temperature of the column was 40 °C; the injection volume was 2 μl and the flow rate was 0.4 ml per minute.

The MS parameter was set as described previously^16,18^. In brief, the temperature of ESI source was 500 °C; the voltage of ion spray was 5500 V; ion source gas I, gas II and curtain gas were set at 55, 60, and 25.0 psi, respectively; the collision gas was set to high. QQQ scans were acquired as MRM experiments with collision gas set to 5 psi. Declustering potential and collision energy for individual MRM transitions was performed with further optimization.

### Qualitative and quantitative analysis of metabolites

Based on the MVDB V2.0 Database of Wuhan Maiteville Biotechnology Co., Ltd. (Wuhan, China) and the metabolite information public database^27–31^, the qualitative analysis was performed according to the secondary spectrum information. The isotopic signal is removed during analysis, including K^+^, Na^+^, NH_4_^+^ and other fragment ions of large molecular weight substances. The quantitative analysis of metabolites was based on the MRM mode as described previously^16,18^. In the MRM mode, the mass spectrum peak of each different color represented a metabolite. The characteristic ions for each metabolite were filtrated through the triple quadrupole mass spectrometer to obtain the signal strengths. Integration of chromatographic peaks was carried out using MultiQuant. In order to ensure the qualitative and quantitative accuracy, the mass spectrum peaks detected in different samples of each metabolite were corrected based on retention time and peak type according to the method described by Fraga *et al*.^32^.

### Statistical analysis

Before analysis, the raw metabolic data were normalized by the method described previously^33^, and the normalized data were log_2_ transformed before they were used for further analysis. Hierarchical clustering analysis (HCA), principle component analysis (PCA) and partial least squares-discriminant analysis (PLS-DA) have been used to analyze the multivariate and differences of metabolites by soft R (www.r-project.org/) according to the previous study described^34,35^. Based the results of PLS-DA, a fold-change ≥2 or ≤0.5 among the metabolites with a VIP (variable importance in project) value >1 was used to identify differential metabolites. The Kyoto Encyclopedia of Genes and Genomes (KEGG) database was used to annotate the differential metabolites and analyze metabolic pathways^36^.

## Conclusions

Wood color is one of the important factors related to wood property. It is critical to understand the chemical compositions that determine the wood color formation. The HW of *T. chinensis* has high commercial value for its purple red brown color and texture density. To the best of our knowledge, the difference of components between the HW and SW in *T. chinensis* have not been previously investigated. In this study, the components of the HW and SW in *T. chinensis* have been analyzed using widely targeted metabolic profiling. A total of 607 metabolites were detected in HW and SW. Among them, 146 metabolites were found significantly higher and 167 metabolites significantly lower in HW as compared to SW. These differential metabolites were mainly involved in metabolic pathways and biosynthesis of secondary metabolites, such as flavonoids, flavone and flavonol, phenylpropanoids and antibiotics. Moreover, the flavonoids in HW associated with wood color were identified. The results provide insight into the metabolites associated with wood color formation and may be useful for understanding the metabolites associated with wood quality.

## Supplementary information

Dataset 1
