# Supplementary material for: A comparative metabolomics analysis of the components of heartwood and sapwood in Taxus chinensis (Pilger) Rehd
Source: Sci Rep. 2019 Nov 27;9:17647. doi: 10.1038/s41598-019-53839-2 (PMC6881444; doi:10.1038/s41598-019-53839-2)
Supplement: Supplementary file 1 — Dataset 1 [file 41598_2019_53839_MOESM1_ESM.pdf]

A comparative metabolomics analysis of the components of heartwood and sapwood in *Taxus chinensis* (Pilger) Rehd.

Fenjuan Shao[1], Lisha Zhang[1], Juan Guo[2], Xiaochun Liu[3], Wenhui Ma[4], Iain W Wilson[5], Deyou Qiu[1]*

1 State Key Laboratory of Tree Genetics and Breeding, Key Laboratory of Tree Breeding and Cultivation of National Forestry and Grassland Administration, Research Institute of Forestry, Chinese Academy of Forestry, Beijing, 100091, China

2 Research Institute of Wood Industry, Chinese Academy of Forestry, Beijing 100091, China

3 Gansu Forestry and Grassland Administration, Lanzhou 730030, China

4 Liangdang Forestry Administration, Gansu Province 742400, China

5 CSIRO Agriculture and Food, PO Box 1700, Canberra, ACT 2601, Australia.

***Corresponding author:** Deyou Qiu. State Key Laboratory of Tree Genetics and Breeding, Key Laboratory of Tree Breeding and Cultivation of National Forestry and Grassland Administration, Research Institute of Forestry, Chinese Academy of Forestry, Beijing, 100091, China. Tel: +86 10 62889641; Fax: +86 10 62882015;

Supplementary file 1: Identified metabolites of the heartwood and sapwood.

Index	Compounds	Class	VIP	Fold_Change	Log2FC
pmf0296	16-Hydroxy hexadecanoic acid	Lipids	1.23	115148.15	16.81
pmb0863	LysoPC 16:2 (2n isomer)	Lipids	1.23	68037.04	16.05
pmb0890	MAG (18:2)	Lipids	1.23	40592.59	15.31
pmb2467	α-Linolenic acid	Lipids	1.23	25481.48	14.64
pmb0149	MAG (18:4) isomer1	Lipids	1.22	14974.07	13.87
pmb2325	MAG (18:3) isomer2	Lipids	1.23	14666.67	13.84
pma1303	LysoPC 16:2	Lipids	1.22	14114.81	13.78
pmb0864	LysoPE 14:0	Lipids	1.22	7188.89	12.81
pmb2787	9-KODE	Lipids	1.23	5255.56	12.36
pmb2640	Lauric acid (C12:0)	Lipids	1.23	1766.67	10.79
pmf0401	Docosanoic acid	Lipids	0.87	811.44	9.66
pmb2221	4-Hydroxysphinganine	Lipids	0.99	18.04	4.17
pmb1605	MAG (18:3) isomer3	Lipids	0.97	17.58	4.14
pmb0165	LysoPC 16:1	Lipids	0.95	14.09	3.82
pmf0396	Linoleic acid	Lipids	1.18	5.50	2.46
pmb0159	DGMG (18:2) isomer2	Lipids	0.71	4.99	2.32
pmb1562	MAG (18:4) isomer3	Lipids	0.62	2.76	1.46
pmb0889	Punicic acid	Lipids	1.15	2.64	1.40
pmb2786	9-HOTrE	Lipids	1.09	2.29	1.19
pma3606	9-Hydroxy-(10E,12Z,15Z)-octadecatrienoic acid	Lipids	0.68	1.67	0.74
pma0461	14,15-Dehydrocrepenynic acid	Lipids	1.02	1.63	0.71
pmf0402	Lignoceric acid	Lipids	0.77	1.48	0.56
pmb2792	13-HOTrE(r)	Lipids	0.60	1.31	0.39
pmb1574	Octadecadien-6-ynoic acid	Lipids	0.63	1.28	0.36
pmb2791	9-HpOTrE	Lipids	0.66	1.22	0.29
pmf0293	1-Octadecanol	Lipids	1.07	1.12	0.17
pmb0865	LysoPC 18:3 (2n isomer)	Lipids	0.24	1.09	0.12
pmb2643	Myristoleic acid (C14:1)	Lipids	0.01	0.99	-0.01
pmf0399	cis-Gondoic acid	Lipids	0.17	0.98	-0.03
pmb2804	13-HPODE	Lipids	0.19	0.96	-0.06
pmb3121	LysoPE 18:1	Lipids	0.49	0.82	-0.29
pmb0292	Cholesterol	Lipids	0.81	0.82	-0.29
pmb3132	LysoPE 18:1 (2n isomer)	Lipids	0.73	0.72	-0.47
pme2827	Palmitaldehyde	Lipids	0.78	0.68	-0.56
pmb0873	LysoPC 18:2 (2n isomer)	Lipids	0.94	0.61	-0.72
pmf0398	Arachidic acid	Lipids	1.19	0.45	-1.14
pmb0882	LysoPC 18:1	Lipids	1.11	0.37	-1.43
pmb1650	Octadeca-11E,13E,15Z-trienoic acic	Lipids	1.21	0.36	-1.48
pmd0160	LysoPE 16:0 (2n isomer)	Lipids	1.16	0.31	-1.67
pmb3117	LysoPE 16:0	Lipids	1.15	0.31	-1.70
pmb0160	MAG (18:3) isomer5	Lipids	0.76	0.13	-2.94
pmc0960	LysoPC 20:4	Lipids	1.21	0.12	-3.01
pmb0859	LysoPC 18:1 (2n isomer)	Lipids	1.23	0.12	-3.08
pmb2388	LysoPC 18:0 (2n isomer)	Lipids	1.00	0.06	-3.99

pmd0136	LysoPC 18:0	Lipids	1.22	0.06	-4.14
pmb2319	LysoPC 15:0	Lipids	0.92	0.04	-4.52
pmb0854	LysoPC 18:3	Lipids	1.22	0.01	-6.50
pmb0855	LysoPC 16:0	Lipids	1.22	0.01	-6.59
pmd0132	LysoPC 16:0 (2n isomer)	Lipids	1.22	0.01	-6.68
pmb2228	LysoPC 19:0	Lipids	1.23	0.00	-13.73
pmf0395	Oleic acid	Lipids	0.86	0.00	-17.63
pmb2406	LysoPC 17:0	Lipids	1.23	0.00	-17.82
pmf0159	Azadiradione	Sterides	1.23	18333.33	14.16
pmf0564	Ecdysterone	Sterides	1.20	7.98	3.00
pmf0073	Campesterol	Sterides	1.18	1.12	0.16
pmf0079	Soyasapogenol B	Sterides	1.06	1.05	0.07
pmf0070	β-Sitosterol	Sterides	0.51	0.90	-0.15
pmf0091	2,3-Oxidosqualene	Sterides	0.91	0.87	-0.20
pmf0085	Cycloartenol	Sterides	0.60	0.86	-0.21
pmf0513	Ruscogenin	Sterides	1.23	0.00	-13.35
pme0237	Ethyl 3,4-Dihydroxybenzoate (Ethyl protocatechuate)	Organic acids and derivatives	1.23	266296.30	18.02
pmb1587	4-Hydroxy-3,5-diisopropylbenzaldehyde	Organic acids and derivatives	1.23	100814.81	16.62
pmb0751	5-O-p-Coumaroyl shikimic acid	Organic acids and derivatives	1.22	29518.52	14.85
pme1724	Methyl benzoate	Organic acids and derivatives	1.22	19970.37	14.29
pmf0419	Salicin	Organic acids and derivatives	1.23	18555.56	14.18
pma0149	Sinapoyl malate	Organic acids and derivatives	0.91	56.00	5.81
pme2362	Mandelic acid	Organic acids and derivatives	1.15	34.29	5.10
pmb0404	Arabidopyl shikimic acid	Organic acids and derivatives	0.98	17.85	4.16
pme0310	Ethyl gallate	Organic acids and derivatives	0.92	9.68	3.27
pme0401	Chlorogenic acid methyl ester	Organic acids and derivatives	0.79	8.72	3.12
pma3724	1-O-Feruloyl quinic acid	Organic acids and derivatives	0.71	5.43	2.44
pmb3074	3-O-p-Coumaroyl quinic acid	Organic acids and derivatives	1.08	5.42	2.44
pme0293	(3,4-Dimethoxyphenyl) acetic acid	Organic acids and derivatives	0.70	5.05	2.34
pme2670	4-Hydroxybenzaldehyde	Organic acids and derivatives	0.68	3.78	1.92
pme1814	2,5-dihydroxybenzoic acid (Gentisic acid)	Organic acids and derivatives	1.18	3.36	1.75
pme3198	2,4-Dihydroxybenzoic acid	Organic acids and derivatives	1.17	3.32	1.73
pme2706	2,3-Dihydroxybenzoic acid	Organic acids and derivatives	1.17	3.31	1.73
pmf0066	2'-Acetylacteoside	Organic acids and derivatives	0.42	3.07	1.62
pme2761	4-Hydroxy-2-oxoglutaric acid	Organic acids and derivatives	1.19	2.87	1.52
pmf0280	D-Galacturonic acid	Organic acids and derivatives	1.20	2.50	1.32
pmb0758	Anisic acid O-feruloyl hexoside	Organic acids and derivatives	1.02	2.44	1.29
pme3085	2-(Formylamino)benzoic acid	Organic acids and derivatives	0.89	2.18	1.12
pme2603	3-Hydroxyanthranilic acid	Organic acids and derivatives	0.48	1.97	0.98
pmf0096	Oxalic acid	Organic acids and derivatives	0.40	1.44	0.53
pme0240	Benzoic acid	Organic acids and derivatives	0.96	1.44	0.53
pme3207	trans,trans-Muconic acid	Organic acids and derivatives	0.80	1.33	0.41
pme0397	Shikimic acid	Organic acids and derivatives	0.18	1.29	0.36
pme3069	Taurocholic acid	Organic acids and derivatives	0.78	1.27	0.34
pmf0408	Plantamajoside	Organic acids and derivatives	0.38	1.26	0.34

pme2131	3-(3-hydroxyphenyl)propionate acid	Organic acids and derivatives	0.37	1.15	0.20
pmb3075	3-O-p-Coumaroyl shikimic acid	Organic acids and derivatives	0.54	1.13	0.18
pme0024	2-Aminoethanesulfonic acid	Organic acids and derivatives	0.12	1.10	0.13
pmb0247	p-Aminobenzoate	Organic acids and derivatives	0.12	1.01	0.01
pmb3099	Diethyl phosphate	Organic acids and derivatives	0.55	0.92	-0.12
pme2598	3,4-Dihydroxybenzeneacetic acid	Organic acids and derivatives	0.28	0.91	-0.14
pme0049	2-Aminoethanesulfinic acid	Organic acids and derivatives	0.60	0.89	-0.16
pme0281	Terephthalic acid	Organic acids and derivatives	0.85	0.89	-0.17
pmb0766	Azoxystrobin acid	Organic acids and derivatives	0.41	0.86	-0.22
pme1292	Homogentisic acid	Organic acids and derivatives	0.65	0.83	-0.27
pme2550	Cis-Aconitic acid	Organic acids and derivatives	0.47	0.82	-0.28
pme1852	Creatine	Organic acids and derivatives	0.49	0.81	-0.30
pme0474	4-Hydroxybenzoic acid	Organic acids and derivatives	1.07	0.67	-0.59
pme0398	Chlorogenic acid (3-O-Caffeoylquinic acid)	Organic acids and derivatives	0.56	0.57	-0.81
pme3009	trans-Citridic acid	Organic acids and derivatives	1.20	0.53	-0.92
pme0269	Dodecanedioic aicd	Organic acids and derivatives	1.13	0.52	-0.93
pme1816	Neochlorogenic acid (5-O-Caffeoylquinic acid)	Organic acids and derivatives	0.57	0.48	-1.04
pme0309	Methyl gallate	Organic acids and derivatives	1.07	0.47	-1.07
pme1162	Gallic acid	Organic acids and derivatives	0.67	0.44	-1.20
pme0239	2–furanoic acid	Organic acids and derivatives	1.09	0.42	-1.24
pme2901	1-O-Caffeoyl quinic acid	Organic acids and derivatives	0.42	0.41	-1.29
pme1730	D-Erythronolactone	Organic acids and derivatives	1.20	0.41	-1.29
pme1820	p–Hydroxyphenyl acetic acid	Organic acids and derivatives	1.10	0.35	-1.50
pme2380	A-Ketoglutaric acid	Organic acids and derivatives	1.21	0.32	-1.62
pme2923	Acetoxyacetic acid	Organic acids and derivatives	1.05	0.32	-1.65
pmf0420	Benzyl acetate	Organic acids and derivatives	0.88	0.29	-1.79
pme2601	3-Hydroxypropanoic acid	Organic acids and derivatives	1.01	0.28	-1.83
pmb2654	Anthranilate O-hexosyl-O-hexoside	Organic acids and derivatives	1.05	0.27	-1.91
pme0486	Methylmalonic acid	Organic acids and derivatives	1.18	0.25	-2.00
pme3096	Aminomalonic acid	Organic acids and derivatives	1.18	0.25	-2.01
pme1830	Succinic acid	Organic acids and derivatives	1.18	0.25	-2.01
pme0291	Dl-2-Aminooctanoic acid	Organic acids and derivatives	1.21	0.24	-2.05
pmb2826	Citramalate	Organic acids and derivatives	1.03	0.21	-2.22
pmf0578	Citric acid monohydrate	Organic acids and derivatives	1.01	0.21	-2.24
pme0413	Vanillin	Organic acids and derivatives	1.21	0.20	-2.33
pme2009	L-(+)-Tartaric acid	Organic acids and derivatives	1.18	0.20	-2.34
pme2241	Citraconic acid	Organic acids and derivatives	1.14	0.17	-2.57
pme2036	Quinic acid	Organic acids and derivatives	1.14	0.14	-2.80
pme0207	3-Hydroxybutyrate	Organic acids and derivatives	1.19	0.14	-2.81
pmf0425	D-tartaric acid	Organic acids and derivatives	1.21	0.13	-2.90
pme3719	D-Xylonic acid	Organic acids and derivatives	1.21	0.13	-2.98
pme2050	Citric acid	Organic acids and derivatives	1.10	0.11	-3.25
pmb1912	10-Formyl-THF	Organic acids and derivatives	1.20	0.10	-3.37
pme2169	Fumaric acid	Organic acids and derivatives	1.20	0.09	-3.48
pme0275	4-Oxopentanoate	Organic acids and derivatives	1.19	0.07	-3.82
pme3346	5-hydroxyhexanoic acid	Organic acids and derivatives	0.85	0.05	-4.21

pme1218	2-Picolinic acid	Organic acids and derivatives	0.89	0.03	-5.03
pme0271	Maleic acid	Organic acids and derivatives	1.05	0.03	-5.20
pme2049	2-Hydroxybutanoic acid	Organic acids and derivatives	1.22	0.02	-5.49
pmb2657	Argininosuccinate	Organic acids and derivatives	1.14	0.02	-5.52
pme2033	L(-)-Malic acid	Organic acids and derivatives	1.22	0.01	-6.24
pme3309	2-Methylglutaric acid	Organic acids and derivatives	1.23	0.01	-6.27
pme3098	4-Methylvaleric acid	Organic acids and derivatives	1.09	0.01	-6.67
pme2541	α-Hydroxyisobutyric acid	Organic acids and derivatives	1.07	0.01	-6.83
pme0243	Glutaric acid	Organic acids and derivatives	1.22	0.01	-7.00
pme2129	(S)-(-)-2-Hydroxyisocaproic acid	Organic acids and derivatives	1.20	0.01	-7.01
pme0286	2-Hydroxyisocaproic acid	Organic acids and derivatives	0.99	0.01	-7.06
pme0267	2-Methylsuccinic acid	Organic acids and derivatives	1.22	0.01	-7.20
pme0250	Azelaic acid	Organic acids and derivatives	1.21	0.01	-7.62
pme1977	Suberic acid	Organic acids and derivatives	1.22	0.00	-8.43
pme2935	2-Oxovaleric acid	Organic acids and derivatives	1.23	0.00	-11.62
pmb2928	Gallic acid O-Hexoside	Organic acids and derivatives	1.23	0.00	-12.04
pme2284	Pentadecafluorooctanoic acid	Organic acids and derivatives	1.23	0.00	-12.78
pme1299	Dl-3,4-Dihydroxymandelic acid	Organic acids and derivatives	1.23	0.00	-13.85
pme1711	3-Hydroxy-3-methyl butyric acid	Organic acids and derivatives	1.23	0.00	-13.87
pme0266	Sebacate	Organic acids and derivatives	1.23	0.00	-14.15
pme3154	(Rs)-Mevalonic acid	Organic acids and derivatives	1.23	0.00	-14.58
pme3034	ethylmalonate	Organic acids and derivatives	1.23	0.00	-16.32
pmb2871	,5-dihydroxy benzoic acid O-hexsid	Organic acids and derivatives	1.23	0.00	-17.28
pme0542	Indole-2-carboxylic acid	Indole derivatives	0.87	4118.85	12.01
pmb0312	5-methoxyindole-3-carbaldehyde	Indole derivatives	0.94	11.11	3.47
pmb0813	1-Methoxyindole-3-carbaldehyde	Indole derivatives	1.15	6.92	2.79
pme2720	Indole-3-carboxaldehyde	Indole derivatives	0.97	0.08	-3.56
pme0543	Indole-5-carboxylic acid	Indole derivatives	0.99	0.04	-4.68
pme3279	2'-Hydroxygenistein	Isoflavone	1.23	45962.96	15.49
pme1578	enistein (4',5,7-Trihydroxyisoflavon	Isoflavone	0.87	43111.44	15.40
pme3451	Rotenone	Isoflavone	1.23	35777.78	15.13
pme3233	Calycosin	Isoflavone	0.95	15.93	3.99
pme3210	Genistein 7-O-Glucoside (Genistin)	Isoflavone	1.21	1.77	0.83
pme1496	Formononetin (4'-O-methyldaidzein	Isoflavone	0.60	0.56	-0.85
pme3292	Prunetin	Isoflavone	1.04	0.32	-1.66
pme3250	Biochanin A	Isoflavone	1.06	0.26	-1.92
pme3263	2'-Hydroxydaidzein	Isoflavone	1.23	0.00	-15.93
pme2289	Vitamin A	Vitamins and derivatives	1.23	314444.44	18.26
pme2266	Biotin	Vitamins and derivatives	1.21	28.20	4.82
pme1952	Riboflavin	Vitamins and derivatives	1.16	8.94	3.16
pme2819	All-trans-13,14-dihydroretinol	Vitamins and derivatives	0.72	7.64	2.93
pma3101	Nicotinate ribonucleoside	Vitamins and derivatives	0.97	1.69	0.75
pme2111	L-ascorbate	Vitamins and derivatives	1.02	1.22	0.29
pme1383	Pyridoxine	Vitamins and derivatives	1.02	0.39	-1.35
pme0491	6-hydroxynicotinic acid	Vitamins and derivatives	0.98	0.37	-1.45
pme0496	Nicotinic acid	Vitamins and derivatives	1.14	0.29	-1.80
pme2167	Orotic acid	Vitamins and derivatives	1.13	0.23	-2.12
pmb0802	D-Pantothenic acid	Vitamins and derivatives	1.00	0.03	-4.91

pme1014	Menaquinone (K2)	Vitamins and derivatives	0.54	0.00	-13.39
pmf0604	β-Caryophyllene	Terpene	1.23	1548148.15	20.56
pmf0476	Crocetin	Terpene	1.23	119666.67	16.87
pmb2222	Phytocassane C	Terpene	1.23	46925.93	15.52
pme0080	Cucurbitacin B	Terpene	1.22	22837.04	14.48
pmb1530	Phytocassane D	Terpene	1.23	21407.41	14.39
pmf0592	Artemisinin	Terpene	1.23	17925.93	14.13
pme2184	Nomilin	Terpene	1.22	14600.00	13.83
pme0064	Cucurbitacin I	Terpene	1.22	4162.96	12.02
pmf0286	Phytol	Terpene	1.20	4.64	2.21
pmf0477	Pachymic acid	Terpene	0.13	1.13	0.17
pmf0509	(-)-Camphor	Terpene	0.63	1.03	0.05
pmf0613	α-Terpineol	Terpene	0.08	1.00	0.00
pme1643	(+)-piperitol	Terpene	0.11	0.96	-0.05
pme0062	Cucurbitacin D	Terpene	0.71	0.73	-0.46
pmf0465	Geniposide	Terpene	0.95	0.57	-0.81
pmf0497	Albiflorin	Terpene	0.61	0.44	-1.18
pmf0325	Glucocheirolin	Terpene	0.90	0.15	-2.69
pmf0272	Galactinol Dihydrate	Carbohydrates	1.23	9485.19	13.21
pmf0574	Sucralose	Carbohydrates	1.06	107.05	6.74
pme1846	D(+)-Glucose	Carbohydrates	1.07	1.50	0.58
pmb2858	Maltotetraose	Carbohydrates	0.63	1.34	0.43
pmf0139	D-(+)-Galactose	Carbohydrates	0.33	0.96	-0.06
pme2435	L-Fucose	Carbohydrates	1.10	0.65	-0.63
pmf0138	D-(+)-Mannose	Carbohydrates	1.18	0.55	-0.86
pme1021	D-(+)-Glucono-1,5-lactone	Carbohydrates	1.18	0.27	-1.87
pme2019	DL-Arabinose	Carbohydrates	1.14	0.21	-2.22
pma6455	Ribulose-5-phosphate	Carbohydrates	0.75	0.16	-2.65
pmf0282	Melibiose	Carbohydrates	1.15	0.13	-2.97
pme0519	D-(+)-Sucrose	Carbohydrates	1.15	0.12	-3.05
pmf0032	Galactinol	Carbohydrates	1.16	0.12	-3.11
pme3313	D-Fructose 6-phosphate	Carbohydrates	1.14	0.07	-3.93
pmb2653	D(+)-Melezitose O-rhamnoside	Carbohydrates	0.92	0.04	-4.61
pme3160	D-Glucose 6-phosphate	Carbohydrates	1.20	0.03	-4.97
pmf0035	Glucose-1-phosphate	Carbohydrates	1.18	0.03	-5.01
pmf0220	)-Fructose 6-phosphate-disodium sa	Carbohydrates	1.19	0.02	-5.36
pmb3088	Trehalose 6-phosphate	Carbohydrates	1.20	0.01	-6.41
pme0500	D(+)-Melezitose	Carbohydrates	0.99	0.01	-6.71
pmf0485	Panose	Carbohydrates	1.21	0.01	-6.95
pmf0481	Dehydrocorydaline	Alkaloids	1.22	786666.67	19.59
pme2658	N-Methyltryptamine	Alkaloids	1.23	165925.93	17.34
pmb2849	Camptothecin	Alkaloids	1.23	47222.22	15.53
pmf0500	Capsaicin	Alkaloids	1.22	43666.67	15.41
pmf0311	Coronatine	Alkaloids	1.22	33703.70	15.04
pmf0328	lumichrome	Alkaloids	1.23	31370.37	14.94
pmf0612	Piperine	Alkaloids	1.23	16222.22	13.99
pmf0307	Solasodine	Alkaloids	1.22	11851.85	13.53
pmf0329	Protopine	Alkaloids	1.22	10151.85	13.31

pmb0426	5-Methoxy-N,N-dimethyltryptamine	Alkaloids	1.13	988.93	9.95
pmf0473	Baccatin III	Alkaloids	1.01	512.73	9.00
pmf0102	Ajmaline	Alkaloids	1.02	434.11	8.76
pme2786	N-Acetyl-5-hydroxytryptamine	Alkaloids	1.05	104.35	6.71
pmf0557	Acid orange 20	Alkaloids	0.83	19.10	4.26
pmb0770	N-Feruloyl serotonin	Alkaloids	0.96	13.04	3.70
pmb0242	Sinapoylcholine	Alkaloids	0.94	9.81	3.29
pma1840	Coumaroyl choline	Alkaloids	0.72	5.33	2.41
pmf0124	2-Hydroxymelatonin	Alkaloids	0.60	2.15	1.10
pmb2211	Cocamidopropyl betaine	Alkaloids	0.49	1.77	0.83
pme2812	6-Hydroxymelatonin	Alkaloids	0.77	1.69	0.76
pmf0565	L-Dencichin	Alkaloids	1.14	1.56	0.64
pmf0213	Caffeine	Alkaloids	0.53	1.47	0.55
pme2268	Trigonelline	Alkaloids	0.24	1.07	0.09
pmf0455	Peimine	Alkaloids	0.46	0.93	-0.10
pme1691	Acetylcholine	Alkaloids	0.35	0.93	-0.10
pmf0531	N-Methylcytisine	Alkaloids	1.13	0.70	-0.51
pmb0782	Piperidine	Alkaloids	0.89	0.63	-0.66
pme1453	Melatonin	Alkaloids	0.48	0.51	-0.98
pmf0384	Nicotine	Alkaloids	0.71	0.31	-1.70
pme2596	4-Pyridoxic acid	Alkaloids	1.17	0.22	-2.19
pmf0436	1-Deoxynojirimycin	Alkaloids	0.75	0.20	-2.36
pmf0267	Indole-3-carboxylic	Alkaloids	0.86	0.05	-4.30
pmf0336	Indole-3-carboxylic acid	Alkaloids	0.86	0.05	-4.37
pmb1754	O-Phosphocholine	Alkaloids	1.13	0.04	-4.52
pma6270	sn-Glycero-3-phosphocholine	Alkaloids	1.21	0.01	-7.03
pma2405	Feruloylcholine	Alkaloids	1.23	0.00	-13.71
pmf0386	Prehelminthosporolactone	Others	1.22	100555.56	16.62
pmf0518	Dehydrocostus lactone	Others	1.23	23000.00	14.49
pmf0437	Harpagoside	Others	1.23	21592.59	14.40
pma3499	Digitoxigenine	Others	1.22	15959.26	13.96
pmf0522	alpha-Santonin	Others	1.23	13337.04	13.70
pmf0516	Mangiferin	Others	0.86	11037.37	13.43
pmf0391	Batatasin III	Others	1.23	6037.04	12.56
pmf0387	Dihydroprehelminthosporol	Others	1.23	5074.07	12.31
pmf0535	Carvacrol	Others	1.23	1469.63	10.52
pmf0580	Carnosol	Others	0.72	8.83	3.14
pme3705	D-glucoronic acid	Others	1.21	3.82	1.93
pmf0443	Parthenolide	Others	0.64	2.93	1.55
pmf0141	2-Phenylethanol	Others	1.04	1.57	0.65
pmf0606	Pectin (Technical Grade)	Others	0.26	1.31	0.39
pmf0506	Ligustilide	Others	0.45	1.29	0.37
pmf0243	Benzyl β-D-Glucopyranoside	Others	1.04	1.03	0.04
pme3221	Isoeugenol	Others	0.25	1.02	0.03
pmb1452	N-Lauryldiethanolamine	Others	0.31	0.87	-0.21
pme2828	4-Nitrophenol	Others	0.75	0.86	-0.21
pmf0534	Hinokitiol	Others	1.04	0.71	-0.50
pmf0349	Vomifoliol	Others	0.41	0.60	-0.73

pme2253	L-Gulonic-γ-lactone	Others	1.11	0.55	-0.87
pme2830	O-Phosphorylethanolamine	Others	0.69	0.26	-1.96
pme2831	1,1-Dimethylbiguanide	Others	0.97	0.07	-3.75
pmf0571	6-Aminopenicillanic acid	Others	0.97	0.06	-4.07
pmb2507	2-Deoxyribose 1-phosphate	Others	0.97	0.06	-4.09
pme0534	Gluconic acid	Others	1.20	0.05	-4.31
pmb3081	Glucarate O-Phosphoric acid	Others	1.02	0.02	-5.64
pmf0217	)-Glucose-6-phosphate disodium sal	Others	1.22	0.01	-6.82
pmb3079	N-Acetylglucosamine 1-phosphate	Others	1.22	0.00	-13.75
pmf0418	Curdione	Others	1.23	0.00	-15.28
pmf0421	Aloin A	Quinones	1.23	6492.59	12.66
pmf0546	Emodin	Quinones	0.47	0.92	-0.11
pmf0554	Parietin	Quinones	0.05	0.60	-0.73
pmf0541	Shikonin	Quinones	1.19	0.28	-1.84
pme1399	Xanthohumol	Flavanone	1.23	7825.93	12.93
pmf0058	4',5,7-Trihydroxyflavanone	Flavanone	0.88	28.35	4.83
pme2957	Naringenin chalcone	Flavanone	1.18	20.49	4.36
pme0376	Naringenin	Flavanone	1.18	17.86	4.16
pme1598	Hesperetin 5-O-glucoside	Flavanone	0.02	1.17	0.23
pme3440	Butein	Flavanone	0.81	1.10	0.14
pma0791	Naringenin O-malonylhexoside	Flavanone	0.04	0.65	-0.63
pme0330	Naringenin 7-O-neohesperidoside (Naringin)	Flavanone	0.74	0.45	-1.15
pme0001	Hesperetin 7-O-neohesperidoside (Neohesperidin)	Flavanone	0.79	0.45	-1.15
pme3461	Homoeriodictyol	Flavanone	1.15	0.34	-1.55
pme2949	Hesperetin 7-rutinoside (Hesperidin	Flavanone	1.09	0.24	-2.07
pme1599	7-O-Methyleriodictyol	Flavanone	1.07	0.23	-2.09
pme3282	Afzelechin (3,5,7,4'-Tetrahydroxyflavan)	Flavanone	1.01	0.03	-5.15
pme3235	Liquiritigenin	Flavanone	1.22	0.00	-10.98
pmf0582	Glabridin	Flavonoid	1.23	48370.37	15.56
pmf0551	Tectochrysin	Flavonoid	1.23	44629.63	15.45
pmf0568	Tectorigenin	Flavonoid	1.23	26111.11	14.67
pmf0246	Orientin	Flavonoid	1.23	23074.07	14.49
pmf0611	Chalcone	Flavonoid	1.22	10122.22	13.31
pmf0493	Puararin	Flavonoid	1.23	4048.15	11.98
pmf0549	Diosmin	Flavonoid	1.23	2848.15	11.48
pmf0393	Persicogenin	Flavonoid	0.95	11.45	3.52
pmf0370	Persicoside	Flavonoid	0.07	2.53	1.34
pmf0471	Apiin	Flavonoid	0.31	1.86	0.89
pmf0372	Pedalitin	Flavonoid	0.81	1.72	0.78
pme1665	sovitexin 7-O-glucoside (Saponarin	Flavonoid	0.15	0.94	-0.09
pmf0362	Hydroxygenkwanin	Flavonoid	0.40	0.67	-0.57
pmf0204	Hyperoside	Flavonoid	0.50	0.51	-0.96
pmf0265	5,7-Dihydroxychromone	Flavonoid	1.09	0.49	-1.03
pmf0208	Isoquercitroside	Flavonoid	0.64	0.33	-1.58
pmf0109	3-O-Acetylpinobanksin	Flavonoid	0.66	0.32	-1.65

pmf0274	Herbacetin	Flavonoid	1.17	0.23	-2.13
pmf0179	Narcissoside	Flavonoid	1.11	0.14	-2.83
pmf0464	Farrerol	Flavonoid	1.09	0.14	-2.84
pmf0374	Isorhamnetin 3-O-glucoside	Flavonoid	1.23	0.00	-14.36
pma6389	Ayanin	Flavonol	1.23	708148.15	19.43
pme1478	Myricetin	Flavonol	1.23	50074.07	15.61
pme3137	3-Hydroxyflavone	Flavonol	1.23	36000.00	15.14
pme2963	Aromadedrin (Dihydrokaempferol)	Flavonol	1.23	35185.19	15.10
pme3372	Rhamnetin (7-O-methxyl quercetin)	Flavonol	1.23	16259.26	13.99
pme2898	Dihydromyricetin	Flavonol	0.97	18.54	4.21
pme1588	Isorhamnetin	Flavonol	1.14	13.00	3.70
pme3288	3,7-Di-O-methylquercetin	Flavonol	1.06	7.16	2.84
pme3514	Morin	Flavonol	0.87	3.37	1.75
pme3404	Syringetin	Flavonol	0.86	2.76	1.46
pme3212	Quercetin 3-O-glucoside (Isotrifoliin	Flavonol	0.54	2.47	1.30
pme3267	Kaempferol 3-O-galactoside (Trifolin	Flavonol	0.97	2.14	1.10
pme1622	aempferol 3-O-glucoside (Astragali	Flavonol	0.89	1.94	0.95
pme3410	Laricitrin	Flavonol	1.04	1.87	0.90
pme0199	Quercetin	Flavonol	0.72	1.40	0.48
pme3129	uercetin 4'-O-glucoside (Spiraeosid	Flavonol	0.06	1.22	0.28
pme1500	Kumatakenin	Flavonol	0.53	0.65	-0.62
pme0197	Quercetin 3-O-rutinoside (Rutin)	Flavonol	0.52	0.60	-0.75
pme3297	Kaempferol 3-O-rhamnoside (Kaempferin)	Flavonol	0.98	0.58	-0.78
pme3468	Kaempferol-3-O-robinoside-7-O-rhamnoside(Robinin)	Flavonol	0.82	0.34	-1.57
pmb3894	Di-O-methylquercetin	Flavonol	1.16	0.32	-1.63
pme0321	Kaempferol 7-O-rhamnoside	Flavonol	1.21	0.21	-2.25
pme1605	Kaempferol 3-O-robinobioside (Biorobin)	Flavonol	1.18	0.19	-2.38
pme0369	empferol 3-O-rutinoside (Nicotiflor	Flavonol	1.15	0.19	-2.40
pme3393	Fustin	Flavonol	1.20	0.19	-2.43
pme1539	Isorhamnetin 3-O-neohesperidoside	Flavonol	1.16	0.14	-2.80
pma6639	Isorhamnetin O-hexoside	Flavonol	1.23	0.00	-14.31
pmb0595	Isorhamnetin 5-O-hexoside	Flavonol	1.23	0.00	-14.51
pmb0675	C-pentosyl-apigenin O-p-coumaroylhexoside	Flavone	1.23	2051851.85	20.97
pma6360	O-methylChrysoeriol 8-C-hexoside	Flavone	1.23	131111.11	17.00
pma6199	Chrysin O-hexoside	Flavone	1.23	119259.26	16.86
pmb0566	Luteolin O-hexosyl-O-pentoside	Flavone	1.22	80074.07	16.29
pma6638	O-methylChrysoeriol 7-O-hexoside	Flavone	1.23	75259.26	16.20
pma0253	O-methylChrysoeriol 5-O-hexoside	Flavone	1.22	69925.93	16.09
pmb0623	6-C-hexosyl chrysoeriol O-hexoside	Flavone	1.23	38962.96	15.25
pmb0674	C-pentosyl apigenin O-salicyloyl hexoside	Flavone	1.22	38629.63	15.24
pmb3046	Tricin 7-O-hexoside	Flavone	1.22	36296.30	15.15
pmb0621	C-hexosyl-isorhamnetin O-hexoside	Flavone	1.23	32814.81	15.00
pmb0358	Selgin O-hexosyl-O-hexoside	Flavone	1.23	25481.48	14.64

pmb3053	Tricin O-eudesmic acid	Flavone	1.22	21555.56	14.40
pmb0724	Tricin O-rhamnoside	Flavone	1.23	12848.15	13.65
pmb3051	Tricin 4'-O-syringyl alcohol	Flavone	0.86	12737.37	13.64
pmb3052	Tricin 4'-O-β-guaiacylglycerol	Flavone	1.21	8200.00	13.00
pme1662	sakuranetin	Flavone	0.87	5926.26	12.53
pmb0571	Apigenin O-hexosyl-O-pentoside	Flavone	1.22	4907.41	12.26
pme1611	Isohemiphloin	Flavone	1.23	3637.04	11.83
pmb0720	Tricin O-malonylhexoside	Flavone	0.87	2618.85	11.35
pmb0580	Chrysin 5-O-glucoside (Toringin)	Flavone	1.03	71.33	6.16
pmb0717	Tricin 5-O-β-guaiacylglycerol	Flavone	1.17	39.99	5.32
pme3473	Butin	Flavone	0.88	26.95	4.75
pmf0057	4,2',4',6'-Tetrahydroxychalcone	Flavone	1.19	23.28	4.54
pme0089	Luteolin	Flavone	0.98	20.48	4.36
pme0324	Chrysin	Flavone	0.97	18.22	4.19
pmb0735	Tricin O-glucuronic acid	Flavone	0.50	10.04	3.33
pma6218	O-methylnaringenin C-pentoside	Flavone	1.21	5.66	2.50
pma6576	Spinacetin	Flavone	0.99	3.00	1.58
pme2457	Luteolin 7-O-glucoside (Cynaroside	Flavone	0.64	2.83	1.50
pmb2981	Apigenin C-hexosyl-O-rutinoside	Flavone	1.06	2.27	1.19
pme0359	Apigenin 5-O-glucoside	Flavone	1.18	1.78	0.84
pmb0605	Apigenin 7-O-glucoside (Cosmosiin	Flavone	1.15	1.77	0.82
pme1518	Nobiletin	Flavone	0.67	1.57	0.65
pmb0676	8-C-hexosyl-chrysoeriol O-feruloylhexoside	Flavone	0.40	1.45	0.53
pmb2850	Tricin	Flavone	0.32	1.37	0.45
pme1550	Tangeretin	Flavone	0.13	1.13	0.17
pmb0678	8-C-hexosyl-apigenin O-feruloylhexoside	Flavone	0.00	1.09	0.12
pmf0011	Apigenin 6,8-C-diglucoside	Flavone	0.01	1.05	0.07
pme0374	Isovitexin	Flavone	0.08	0.99	-0.02
pmb0624	6-C-hexosyl-luteolin O-hexoside	Flavone	0.33	0.82	-0.29
pma3443	Tricin 7-O-acetylglucoside	Flavone	0.87	0.64	-0.64
pmb0628	Eriodictiol C-hexosyl-O-hexoside	Flavone	0.03	0.55	-0.87
pmf0005	Narirutin	Flavone	0.78	0.51	-0.97
pma6558	Velutin	Flavone	0.84	0.48	-1.05
pma1108	Apigenin C-glucoside	Flavone	0.60	0.44	-1.17
pme1541	Acacetin	Flavone	0.99	0.42	-1.24
pma6496	Luteolin 6-C-glucoside	Flavone	0.63	0.37	-1.45
pme0363	Chrysoeriol	Flavone	1.10	0.36	-1.47
pme0379	Apigenin	Flavone	1.20	0.30	-1.72
pme3300	Tricetin	Flavone	1.15	0.20	-2.29
pmb0588	Luteolin 3',7-di-O-glucoside	Flavone	1.23	0.00	-12.42
pma0294	Chrysoeriol 5-O-hexoside	Flavone	1.23	0.00	-14.29
pmb0607	Chrysoeriol 7-O-hexoside	Flavone	1.23	0.00	-14.34
pmb0696	8-C-hexosyl chrysoeriol O-hexoside	Flavone	1.23	0.00	-15.69
pme0444	Malvidin 3-O-glucoside (Oenin)	Anthocyanins	1.23	130888.89	17.00
pme0443	Malvidin 3-O-galactoside	Anthocyanins	1.22	101592.59	16.63
pmb0550	Cyanidin 3-O-glucoside (Kuromanin	Anthocyanins	0.88	1.56	0.64

pme3256	elphinidin 3-O-rutinoside (Tulipani	Anthocyanins	0.55	1.55	0.63
pme1397	Pelargonidin	Anthocyanins	0.56	0.57	-0.81
pmb0542	Cyanidin 3-O-malonylhexoside	Anthocyanins	1.23	0.00	-14.63
pme2776	2'-Deoxyinosine	Nucleotide and derivates	1.23	35296.30	15.11
pme0256	Xanthine	Nucleotide and derivates	1.21	20.14	4.33
pmc0281	Adenosine O-ribose	Nucleotide and derivates	1.22	8.86	3.15
pme3965	2-(dimethylamino)guanosine	Nucleotide and derivates	1.13	3.80	1.93
pme1376	2'-Deoxycytidine-5'-monophosphate	Nucleotide and derivates	0.59	2.09	1.06
pme0130	3-Methylxanthine	Nucleotide and derivates	0.99	1.40	0.48
pme2798	7-Methylxanthine	Nucleotide and derivates	0.81	1.36	0.44
pme1119	Inosine	Nucleotide and derivates	0.57	1.15	0.21
pme0257	Uracil	Nucleotide and derivates	0.02	1.00	0.01
pme0108	5-Methylcytosine	Nucleotide and derivates	0.38	0.75	-0.41
pmc0274	6-Methylmercaptopurine	Nucleotide and derivates	0.95	0.70	-0.52
pme3200	1-methylguanidine	Nucleotide and derivates	1.16	0.69	-0.53
pmc0066	2'-Deoxyinosine-5'-monophosphate	Nucleotide and derivates	1.04	0.50	-1.00
pme0038	Cytosine	Nucleotide and derivates	1.12	0.42	-1.24
pmb0964	iP7G	Nucleotide and derivates	1.20	0.35	-1.53
pmb2922	Uridine 5'-diphospho-D-glucose	Nucleotide and derivates	1.20	0.20	-2.29
pmc0304	Succinyladenosine	Nucleotide and derivates	1.08	0.20	-2.33
pme3336	N6-Succinyl Adenosine	Nucleotide and derivates	1.11	0.19	-2.40
pme3188	Uridine 5'-monophosphate	Nucleotide and derivates	1.19	0.17	-2.52
pmf0059	UDP-α-D-glucose	Nucleotide and derivates	1.21	0.17	-2.59
pme1097	Adenine	Nucleotide and derivates	1.22	0.16	-2.60
pme3961	Deoxyadenosine	Nucleotide and derivates	0.94	0.16	-2.65
pmf0118	dine 5'-diphosphoglucose disodium	Nucleotide and derivates	1.22	0.14	-2.82
pme0183	2-Hydroxy-6-aminopurine	Nucleotide and derivates	1.20	0.14	-2.89
pme1109	Guanine	Nucleotide and derivates	1.20	0.13	-2.92
pme0163	β-Nicotinamide mononucleotide	Nucleotide and derivates	0.96	0.11	-3.20
pme0033	Hypoxanthine	Nucleotide and derivates	1.22	0.09	-3.51
pme3835	;uanosine 3',5'-cyclic monophosphat	Nucleotide and derivates	0.97	0.07	-3.94
pme2282	Guanosine monophosphate	Nucleotide and derivates	0.99	0.06	-4.12
pmb0981	Adenosine 5'-monophosphate	Nucleotide and derivates	1.21	0.03	-5.08
pme3732	Cytidine	Nucleotide and derivates	1.00	0.03	-5.09
pmb0514	Adenosine 3'-monophosphate	Nucleotide and derivates	1.22	0.03	-5.31
pme1175	Guanosine	Nucleotide and derivates	1.03	0.02	-5.45
pmb4344	Guanosine 5'-monophosphate	Nucleotide and derivates	1.06	0.02	-5.71
pme1063	Uridine	Nucleotide and derivates	1.19	0.02	-5.76
pmb0532	Inosine 5'-monophosphate	Nucleotide and derivates	1.06	0.01	-6.52
pme1474	5'-Deoxy-5'-(methylthio)adenosine	Nucleotide and derivates	0.87	0.00	-11.84
pme3983	ypoxanthine-9-β-D-arabinofuranosi	Nucleotide and derivates	0.86	0.00	-13.14
pmd0023	Adenosine	Nucleotide and derivates	1.23	0.00	-13.61
pme2879	5-Hydroxymethyluracil	Nucleotide and derivates	1.23	0.00	-14.01
pme3174	idine 5'-monophosphate (Cytidylic a	Nucleotide and derivates	1.22	0.00	-14.58
pme1294	Xanthosine	Nucleotide and derivates	1.23	0.00	-14.67
pme3197	Cyclic AMP	Nucleotide and derivates	1.22	0.00	-16.33
pmb0130	N-Acetyl tryptamine	Phenolamides	0.89	59.70	5.90
pma1839	N-sinapoyl cadaverine	Phenolamides	1.03	54.94	5.78

pmb0496	N-Feruloyl agmatine	Phenolamides	0.64	2.76	1.46
pmb0493	N'-p-Coumaroyl agmatine	Phenolamides	0.03	1.03	0.04
pmb0501	Agmatine	Phenolamides	0.38	0.95	-0.08
pmd0017	Spermine	Phenolamides	1.21	0.30	-1.71
pmb0488	Spermidine	Phenolamides	1.15	0.26	-1.93
pme2247	Ellagic acid	Polyphenol	1.22	162481.48	17.31
pmf0453	Demethoxycurcumin	Polyphenol	1.23	17407.41	14.09
pme2478	Protocatechuic aldehyde	Polyphenol	1.13	3.33	1.74
pme1824	Protocatechuic acid	Polyphenol	1.17	3.31	1.73
pme0450	L-Epicatechin	Polyphenol	0.18	0.88	-0.19
pme0205	Catechin	Polyphenol	0.34	0.68	-0.56
pme1564	Epicatechin gallate (ECG)	Polyphenol	0.33	0.62	-0.69
pme0426	4-Methylcatechol	Polyphenol	0.92	0.22	-2.16
pmf0458	6-Gingerol	Polyphenol	0.94	0.07	-3.89
pmb2831	Protocatechuic acid O-glucoside	Polyphenol	1.22	0.00	-17.62
pmf0122	1,10-decanediol	Alcohols	1.22	59000.00	15.85
pmb0767	D-erythro-Dihydrosphingosine	Alcohols	1.22	28592.59	14.80
pme0516	Inositol	Alcohols	1.22	7.30	2.87
pme2237	Dulcitol	Alcohols	1.16	2.69	1.43
pme0499	D-Sorbitol	Alcohols	1.14	2.68	1.42
pme0513	Xylitol	Alcohols	0.99	1.84	0.88
pme2134	DL-threitol	Alcohols	0.03	1.16	0.21
pmf0256	3-Methyl-1-pentanol	Alcohols	0.24	0.96	-0.06
pme2256	D-Arabitol	Alcohols	0.70	0.80	-0.33
pme1261	Pantothenol	Alcohols	0.66	0.79	-0.33
pmf0388	Dehydrovomifoliol	Alcohols	0.97	0.45	-1.15
pma2987	Histidinol	Alcohols	1.18	0.42	-1.25
pme2636	Enterodiol	Alcohols	1.21	0.39	-1.37
pme2529	1,5-Anhydro-D-glucitol	Alcohols	1.15	0.34	-1.55
pme1944	D-Mannitol	Alcohols	1.20	0.19	-2.36
pmf0175	2-Decanol	Alcohols	1.22	0.12	-3.02
pmf0174	1-Decanol	Alcohols	1.22	0.12	-3.03
pmf0068	Mannitol	Alcohols	0.79	0.10	-3.30
pmf0348	2,6-Dimethyl-7-octene-2,3,6-triol	Alcohols	1.22	0.00	-7.64
pmf0173	1,2-Decanediol	Alcohols	1.23	0.00	-18.44
pmf0010	Xanthotoxol	Phenylpropanoids	1.23	555925.93	19.08
pmf0164	Schizandrin B	Phenylpropanoids	1.23	200740.74	17.61
pmf0404	Ethyl cinnamate	Phenylpropanoids	1.23	81074.07	16.31
pmf0479	8-Methoxypsoralen	Phenylpropanoids	1.23	54333.33	15.73
pme3553	Psoralen	Phenylpropanoids	1.22	51148.15	15.64
pme2987	3,4-Dihydrocoumarin	Phenylpropanoids	1.22	39407.41	15.27
pme1458	Resveratrol	Phenylpropanoids	1.23	20925.93	14.35
pmb0382	O-Feruloyl 4-hydroxylcoumarin	Phenylpropanoids	1.23	19629.63	14.26
pmf0525	Imperatorin	Phenylpropanoids	1.23	18629.63	14.19
pmf0526	Isoimperatorin	Phenylpropanoids	1.23	16851.85	14.04
pmb1178	O-Feruloyl 2-hydroxylcoumarin	Phenylpropanoids	1.23	16222.22	13.99
pme1472	Vanillic acid	Phenylpropanoids	1.23	14666.67	13.84
pmf0527	Osthole	Phenylpropanoids	1.22	14318.52	13.81

pme0299	trans-Cinnamate	Phenylpropanoids	1.23	12222.22	13.58
pmf0543	Podophyllotoxin	Phenylpropanoids	1.01	61.22	5.94
pmf0563	Magnolol	Phenylpropanoids	0.85	40.94	5.36
pmf0320	Acetosyringone	Phenylpropanoids	1.00	27.99	4.81
pme0429	3,4,5-Trimethoxycinnamic acid	Phenylpropanoids	0.95	11.76	3.56
pme3453	p-Coumaraldehyde	Phenylpropanoids	1.18	8.36	3.06
pme0418	3-(4-Hydroxyphenyl)propionic acid	Phenylpropanoids	0.76	7.97	2.99
pme3305	p-Coumaryl alcohol	Phenylpropanoids	1.11	7.57	2.92
pmf0270	(E)-p-coumaric acid	Phenylpropanoids	1.17	7.55	2.92
pme0303	Caffeate	Phenylpropanoids	1.20	7.49	2.90
pme3422	6-Methoxy-7,8-DihydroxyCoumarir	Phenylpropanoids	0.76	6.77	2.76
pmf0165	Schisandrin C	Phenylpropanoids	1.20	5.36	2.42
pmf0586	Schizandrin C	Phenylpropanoids	1.18	4.87	2.28
pme1436	p-Coumaric acid	Phenylpropanoids	1.19	4.22	2.08
pmb0108	Feruloyl syringic acid	Phenylpropanoids	0.64	4.07	2.02
pmf0298	Pyrocatechol	Phenylpropanoids	0.80	2.35	1.23
pmf0354	Catechol	Phenylpropanoids	0.76	2.25	1.17
pmf0062	Verbascoside	Phenylpropanoids	0.14	2.17	1.12
pme3143	6-Hydroxy-4-methylcoumarin	Phenylpropanoids	0.83	1.74	0.80
pmf0067	Isoacteoside	Phenylpropanoids	0.31	1.74	0.80
pme3242	Medicarpin	Phenylpropanoids	0.37	1.26	0.33
pme1638	sesamolin	Phenylpropanoids	1.11	1.04	0.06
pme1637	Coniferyl alcohol	Phenylpropanoids	0.16	1.04	0.05
pmb2835	Syringaldehyde	Phenylpropanoids	0.32	0.92	-0.11
pmf0605	Notopterol	Phenylpropanoids	0.78	0.80	-0.32
pmf0609	Umbelliferone	Phenylpropanoids	0.62	0.63	-0.67
pme3564	Daphnetin	Phenylpropanoids	1.07	0.60	-0.74
pmf0284	-Hydroxy-3-methoxycinnamaldehyc	Phenylpropanoids	1.01	0.56	-0.83
pme0305	Ferulic acid	Phenylpropanoids	0.99	0.48	-1.06
pme0422	3-Hydroxy-4-methoxycinnamic acic	Phenylpropanoids	1.00	0.46	-1.13
pme3419	Esculetin (6,7-dihydroxycoumarin)	Phenylpropanoids	1.21	0.40	-1.33
pmf0572	Paeonol	Phenylpropanoids	1.00	0.40	-1.33
pme1646	Pinoresinol	Phenylpropanoids	0.38	0.39	-1.37
pme2996	4-Hydroxycoumarin	Phenylpropanoids	0.86	0.35	-1.51
pme3131	6,7-Dimethoxy-4-methylcoumarin	Phenylpropanoids	0.63	0.31	-1.67
pme1695	Sinapic acid	Phenylpropanoids	0.71	0.22	-2.15
pme0387	Homovanillic acid	Phenylpropanoids	1.18	0.21	-2.26
pme3443	Sinapinaldehyde	Phenylpropanoids	1.20	0.12	-3.09
pmf0326	Apocynin	Phenylpropanoids	1.03	0.01	-6.18
pmf0456	8-GINGEROL	Phenylpropanoids	0.86	0.00	-8.28
pmf0098	trans-4-Hydroxycinnamic acid Methyl Ester	Phenylpropanoids	1.23	0.00	-12.76
pme3246	Coniferin	Phenylpropanoids	0.86	0.00	-13.33
pme0391	4-Methylumbelliferone	Phenylpropanoids	1.23	0.00	-16.80
pmf0292	D-erythro-sphinganine	Amino acid and derivatives	1.23	26555.56	14.70
pme0195	L-Cysteine	Amino acid and derivatives	1.21	5.25	2.39
pmf0442	N-Acetyl-L-phenylalanine	Amino acid and derivatives	0.70	3.65	1.87
pme2758	4-Hydroxy-L-glutamic acid	Amino acid and derivatives	0.60	2.68	1.42

pmf0588	Aspartic acid	Amino acid and derivatives	0.75	1.81	0.86
pmf0585	Proline	Amino acid and derivatives	0.36	1.36	0.44
pme0122	N6-Acetyl-L-lysine	Amino acid and derivatives	0.49	1.36	0.44
pmb2873	3-(2-Naphthyl)-D-alanine	Amino acid and derivatives	0.40	1.35	0.43
pme0109	(-)-3-(3,4-Dihydroxyphenyl) -2-methylalanine	Amino acid and derivatives	0.53	1.27	0.35
pme0037	L-Histidine	Amino acid and derivatives	0.50	1.19	0.26
pme0009	L-Serine	Amino acid and derivatives	0.07	1.05	0.07
pme0020	L-Phenylalanine	Amino acid and derivatives	0.02	1.02	0.02
pme0128	D-Alanyl-D-Alanine	Amino acid and derivatives	0.04	0.99	-0.01
pme2527	L(+)-Ornithine	Amino acid and derivatives	0.01	0.99	-0.02
pme0015	L-(-)-Cystine	Amino acid and derivatives	0.17	0.98	-0.03
pme0007	L-Citrulline	Amino acid and derivatives	0.05	0.96	-0.06
pme0022	L-Threonine	Amino acid and derivatives	0.26	0.93	-0.10
pme2790	N-γ-Acetyl-N-2-Formyl-5-methoxykynurenamine	Amino acid and derivatives	0.11	0.90	-0.16
pme0226	L-Asparagine	Amino acid and derivatives	0.34	0.90	-0.16
pme0075	N-Acetyl-L-glutamic acid	Amino acid and derivatives	0.66	0.87	-0.20
pmf0594	D-(+)-Phenylalanine	Amino acid and derivatives	0.46	0.86	-0.22
pme1239	S-(methyl)glutathione	Amino acid and derivatives	0.58	0.83	-0.27
pme0042	L-(+)-Arginine	Amino acid and derivatives	0.93	0.75	-0.42
pme0030	L-(-)-Tyrosine	Amino acid and derivatives	0.70	0.73	-0.46
pme0050	L-Tryptophan	Amino acid and derivatives	0.75	0.71	-0.49
pme1712	L-Saccharopine	Amino acid and derivatives	0.95	0.67	-0.58
pme0011	L-Aspartic acid	Amino acid and derivatives	0.74	0.67	-0.58
pme0026	L-(+)-Lysine	Amino acid and derivatives	1.09	0.63	-0.67
pme0013	L-Glutamic acid	Amino acid and derivatives	0.88	0.59	-0.76
pme2122	Histamine	Amino acid and derivatives	0.91	0.58	-0.78
pme0161	L-Homoserine	Amino acid and derivatives	1.11	0.58	-0.80
pmf0589	Glutamic acid	Amino acid and derivatives	0.93	0.55	-0.85
pme1872	L-Proline	Amino acid and derivatives	1.15	0.54	-0.90
pme1368	L-Pipecolic acid	Amino acid and derivatives	0.94	0.52	-0.94
pme1408	L-Glutamine	Amino acid and derivatives	1.12	0.52	-0.95
pme0279	2,6-Diaminooimelic acid	Amino acid and derivatives	1.15	0.47	-1.08
pme2617	Methionine sulfoxide	Amino acid and derivatives	1.23	0.36	-1.46
pmb2857	L-Glutamic acid O-glucoside	Amino acid and derivatives	1.19	0.34	-1.57
pmb2855	L-Glutamine O-hexside	Amino acid and derivatives	1.22	0.30	-1.74
pme3382	N-Acetylthreonine	Amino acid and derivatives	0.67	0.29	-1.77
pme0137	Nα-Acetyl-L-glutamine	Amino acid and derivatives	1.19	0.27	-1.89
pmb0962	Lysine butyrate	Amino acid and derivatives	1.04	0.11	-3.16
pme2634	Dl-Norvaline	Amino acid and derivatives	0.95	0.10	-3.26
pme0039	L-Valine	Amino acid and derivatives	0.95	0.09	-3.43
pme0116	L-Carnosine	Amino acid and derivatives	0.83	0.08	-3.67
pmb0449	2-Aminoadipic acid (L-Homoglutamic acid)	Amino acid and derivatives	0.99	0.04	-4.68
pme2914	3-Hydroxy-3-methylpentane-1,5-dioic acid	Amino acid and derivatives	0.99	0.04	-4.74
pme0004	L-Homocitrulline	Amino acid and derivatives	1.03	0.02	-5.58

pmb3264	Glutathione oxidized	Amino acid and derivatives	1.05	0.02	-5.62
pme0056	2,3-dimethylsuccinic acid	Amino acid and derivatives	1.23	0.01	-6.22
pmf0018	1-Aminocyclopropanecarboxylic acid	Amino acid and derivatives	0.55	0.00	-11.21
pme0173	N-Propionylglycine	Amino acid and derivatives	1.23	0.00	-13.08
pme0180	1-Methylhistidine	Amino acid and derivatives	1.23	0.00	-14.10
pme1322	Nα-Acetyl-L-arginine	Amino acid and derivatives	1.23	0.00	-14.38
pme3827	3,4-Dihydroxy-DL-phenylalanine	Amino acid and derivatives	1.23	0.00	-16.93

Supplementary file 2: The differential metabolites of the heartwood and sapwood.

Index	Compounds	Class	VIP	Fold_Change	Log2FC	Type
pmb0675	C-pentosyl-apigenin O-p-coumaroylhexoside	Flavone	1.23	2051851.85	20.97	up
pmf0604	β-Caryophyllene	Terpene	1.23	1548148.15	20.56	up
pmf0481	Dehydrocorydaline	Alkaloids	1.22	786666.67	19.59	up
pma6389	Ayanin	Flavonol	1.23	708148.15	19.43	up
pmf0010	Xanthotoxol	Phenylpropanoids	1.23	555925.93	19.08	up
pme2289	Vitamin A	Vitamins and derivatives	1.23	314444.44	18.26	up
pme0237	Ethyl 3,4-Dihydroxybenzoate (Ethyl protocatechuate)	Organic acids and derivatives	1.23	266296.30	18.02	up
pmf0164	Schizandrin B	Phenylpropanoids	1.23	200740.74	17.61	up
pme2658	N-Methyltryptamine	Alkaloids	1.23	165925.93	17.34	up
pme2247	Ellagic acid	Polyphenol	1.22	162481.48	17.31	up
pma6360	O-methylChrysoeriol 8-C-hexoside	Flavone	1.23	131111.11	17.00	up
pme0444	Malvidin 3-O-glucoside (Oenin)	Anthocyanins	1.23	130888.89	17.00	up
pmf0476	Crocetin	Terpene	1.23	119666.67	16.87	up
pma6199	Chrysin O-hexoside	Flavone	1.23	119259.26	16.86	up
pmf0296	16-Hydroxy hexadecanoic acid	Lipids	1.23	115148.15	16.81	up
pme0443	Malvidin 3-O-galactoside	Anthocyanins	1.22	101592.59	16.63	up
pmb1587	4-Hydroxy-3,5-diisopropyl benzaldehyde	Organic acids and derivatives	1.23	100814.81	16.62	up
pmf0386	Prehelminthosporolactone	Others	1.22	100555.56	16.62	up
pmf0404	Ethyl cinnamate	Phenylpropanoids	1.23	81074.07	16.31	up
pmb0566	Luteolin O-hexosyl-O-pentoside	Flavone	1.22	80074.07	16.29	up
pma6638	O-methylChrysoeriol 7-O-hexoside	Flavone	1.23	75259.26	16.20	up
pma0253	O-methylChrysoeriol 5-O-hexoside	Flavone	1.22	69925.93	16.09	up
pmb0863	LysoPC 16:2 (2n isomer)	Lipids	1.23	68037.04	16.05	up
pmf0122	1,10-decanediol	Alcohols	1.22	59000.00	15.85	up
pmf0479	8-Methoxypsoralen	Phenylpropanoids	1.23	54333.33	15.73	up
pme3553	Psoralen	Phenylpropanoids	1.22	51148.15	15.64	up
pme1478	Myricetin	Flavonol	1.23	50074.07	15.61	up
pmf0582	Glabridin	Flavonoid	1.23	48370.37	15.56	up
pmb2849	Camptothecin	Alkaloids	1.23	47222.22	15.53	up
pmb2222	Phytocassane C	Terpene	1.23	46925.93	15.52	up
pme3279	2'-Hydroxygenistein	Isoflavone	1.23	45962.96	15.49	up
pmf0551	Tectochrysin	Flavonoid	1.23	44629.63	15.45	up
pmf0500	Capsaicin	Alkaloids	1.22	43666.67	15.41	up
pmb0890	MAG (18:2)	Lipids	1.23	40592.59	15.31	up
pme2987	3,4-Dihydrocoumarin	Phenylpropanoids	1.22	39407.41	15.27	up
pmb0623	6-C-hexosyl chrysoeriol O-hexoside	Flavone	1.23	38962.96	15.25	up
pmb0674	C-pentosyl apigenin O-salicyloyl hexoside	Flavone	1.22	38629.63	15.24	up

pmb3046	Tricin 7-O-hexoside	Flavone	1.22	36296.30	15.15	up
pme3137	3-Hydroxyflavone	Flavonol	1.23	36000.00	15.14	up
pme3451	Rotenone	Isoflavone	1.23	35777.78	15.13	up
pme2776	2'-Deoxyinosine	Nucleotide and derivates	1.23	35296.30	15.11	up
pme2963	Aromadedrin (Dihydrokaempferol)	Flavonol	1.23	35185.19	15.10	up
pmf0311	Coronatine	Alkaloids	1.22	33703.70	15.04	up
pmb0621	C-hexosyl-isorhamnetin O-hexoside	Flavone	1.23	32814.81	15.00	up
pmf0328	lumichrome	Alkaloids	1.23	31370.37	14.94	up
pmb0751	5-O-p-Coumaroyl shikimic acid	Organic acids and derivatives	1.22	29518.52	14.85	up
pmb0767	D-erythro-Dihydrosphingosine	Alcohols	1.22	28592.59	14.80	up
pmf0292	D-erythro-sphinganine	Amino acid and derivatives	1.23	26555.56	14.70	up
pmf0568	Tectorigenin	Flavonoid	1.23	26111.11	14.67	up
pmb0358	Selgin O-hexosyl-O-hexoside	Flavone	1.23	25481.48	14.64	up
pmb2467	α-Linolenic acid	Lipids	1.23	25481.48	14.64	up
pmf0246	Orientin	Flavonoid	1.23	23074.07	14.49	up
pmf0518	Dehydrocostus lactone	Others	1.23	23000.00	14.49	up
pme0080	Cucurbitacin B	Terpene	1.22	22837.04	14.48	up
pmf0437	Harpagoside	Others	1.23	21592.59	14.40	up
pmb3053	Tricin O-eudesmic acid	Flavone	1.22	21555.56	14.40	up
pmb1530	Phytocassane D	Terpene	1.23	21407.41	14.39	up
pme1458	Resveratrol	Phenylpropanoids	1.23	20925.93	14.35	up
pme1724	Methyl benzoate	Organic acids and derivatives	1.22	19970.37	14.29	up
pmb0382	O-Feruloyl 4-hydroxylcoumarin	Phenylpropanoids	1.23	19629.63	14.26	up
pmf0525	Imperatorin	Phenylpropanoids	1.23	18629.63	14.19	up
pmf0419	Salicin	Organic acids and derivatives	1.23	18555.56	14.18	up
pmf0159	Azadiradione	Sterides	1.23	18333.33	14.16	up
pmf0592	Artemisinin	Terpene	1.23	17925.93	14.13	up
pmf0453	Demethoxycurcumin	Polyphenol	1.23	17407.41	14.09	up
pmf0526	Isoimperatorin	Phenylpropanoids	1.23	16851.85	14.04	up
pme3372	Rhamnetin (7-O-methxyl quercetin)	Flavonol	1.23	16259.26	13.99	up
pmb1178	O-Feruloyl 2-hydroxylcoumarin	Phenylpropanoids	1.23	16222.22	13.99	up
pmf0612	Piperine	Alkaloids	1.23	16222.22	13.99	up
pma3499	Digitoxigenine	Others	1.22	15959.26	13.96	up
pmb0149	MAG (18:4) isomer1	Lipids	1.22	14974.07	13.87	up
pmb2325	MAG (18:3) isomer2	Lipids	1.23	14666.67	13.84	up
pme1472	Vanillic acid	Phenylpropanoids	1.23	14666.67	13.84	up
pme2184	Nomilin	Terpene	1.22	14600.00	13.83	up
pmf0527	Osthole	Phenylpropanoids	1.22	14318.52	13.81	up
pma1303	LysoPC 16:2	Lipids	1.22	14114.81	13.78	up
pmf0522	alpha-Santonin	Others	1.23	13337.04	13.70	up
pmb0724	Tricin O-rhamnoside	Flavone	1.23	12848.15	13.65	up
pme0299	trans-Cinnamate	Phenylpropanoids	1.23	12222.22	13.58	up
pmf0307	Solasodine	Alkaloids	1.22	11851.85	13.53	up
pmf0329	Protopine	Alkaloids	1.22	10151.85	13.31	up
pmf0611	Chalcone	Flavonoid	1.22	10122.22	13.31	up

pmf0272	Galactinol Dihydrate	Carbohydrates	1.23	9485.19	13.21	up
pmb3052	Tricin 4'-O-β-guaiacylglycerol	Flavone	1.21	8200.00	13.00	up
pme1399	Xanthohumol	Flavanone	1.23	7825.93	12.93	up
pmb0864	LysoPE 14:0	Lipids	1.22	7188.89	12.81	up
pmf0421	Aloin A	Quinones	1.23	6492.59	12.66	up
pmf0391	Batatasin III	Others	1.23	6037.04	12.56	up
pmb2787	9-KODE	Lipids	1.23	5255.56	12.36	up
pmf0387	Dihydroprehelminthosporol	Others	1.23	5074.07	12.31	up
pmb0571	Apigenin O-hexosyl-O-pentoside	Flavone	1.22	4907.41	12.26	up
pme0064	Cucurbitacin I	Terpene	1.22	4162.96	12.02	up
pmf0493	Puararin	Flavonoid	1.23	4048.15	11.98	up
pme1611	Isohemiphloin	Flavone	1.23	3637.04	11.83	up
pmf0549	Diosmin	Flavonoid	1.23	2848.15	11.48	up
pmb2640	Lauric acid (C12:0)	Lipids	1.23	1766.67	10.79	up
pmf0535	Carvacrol	Others	1.23	1469.63	10.52	up
pmb0426	5-Methoxy-N,N-dimethyltryptamine	Alkaloids	1.13	988.93	9.95	up
pmf0473	Baccatin III	Alkaloids	1.01	512.73	9.00	up
pmf0102	Ajmaline	Alkaloids	1.02	434.11	8.76	up
pmf0574	Sucralose	Carbohydrates	1.06	107.05	6.74	up
pme2786	N-Acetyl-5-hydroxytryptamine	Alkaloids	1.05	104.35	6.71	up
pmb0580	Chrysin 5-O-glucoside (Toringin)	Flavone	1.03	71.33	6.16	up
pmf0543	Podophyllotoxin	Phenylpropanoids	1.01	61.22	5.94	up
pma1839	N-sinapoyl cadaverine	Phenolamides	1.03	54.94	5.78	up
pmb0717	Tricin 5-O-β-guaiacylglycerol	Flavone	1.17	39.99	5.32	up
pme2362	Mandelic acid	Organic acids and derivatives	1.15	34.29	5.10	up
pme2266	Biotin	Vitamins and derivatives	1.21	28.20	4.82	up
pmf0057	4,2',4',6'-Tetrahydroxychalcone	Flavone	1.19	23.28	4.54	up
pme2957	Naringenin chalcone	Flavanone	1.18	20.49	4.36	up
pme0256	Xanthine	Nucleotide and derivates	1.21	20.14	4.33	up
pme0376	Naringenin	Flavanone	1.18	17.86	4.16	up
pme1588	Isorhamnetin	Flavonol	1.14	13.00	3.70	up
pme1952	Riboflavin	Vitamins and derivatives	1.16	8.94	3.16	up
pmc0281	Adenosine O-ribose	Nucleotide and derivates	1.22	8.86	3.15	up
pme3453	p-Coumaraldehyde	Phenylpropanoids	1.18	8.36	3.06	up
pmf0564	Ecdysterone	Sterides	1.20	7.98	3.00	up
pme3305	p-Coumaryl alcohol	Phenylpropanoids	1.11	7.57	2.92	up
pmf0270	(E)-p-coumaric acid	Phenylpropanoids	1.17	7.55	2.92	up
pme0303	Caffeate	Phenylpropanoids	1.20	7.49	2.90	up
pme0516	Inositol	Alcohols	1.22	7.30	2.87	up
pme3288	3,7-Di-O-methylquercetin	Flavonol	1.06	7.16	2.84	up
pmb0813	1-Methoxyindole-3-carbaldehyde	Indole derivatives	1.15	6.92	2.79	up
pma6218	O-methylnaringenin C-pentoside	Flavone	1.21	5.66	2.50	up
pmf0396	Linoleic acid	Lipids	1.18	5.50	2.46	up
pmb3074	3-O-p-Coumaroyl quinic acid	Organic acids and derivatives	1.08	5.42	2.44	up
pmf0165	Schisandrin C	Phenylpropanoids	1.20	5.36	2.42	up
pme0195	L-Cysteine	Amino acid and derivatives	1.21	5.25	2.39	up
pmf0586	Schizandrin C	Phenylpropanoids	1.18	4.87	2.28	up

pmf0286	Phytol	Terpene	1.20	4.64	2.21	up
pme1436	p-Coumaric acid	Phenylpropanoids	1.19	4.22	2.08	up
pme3705	D-glucoronic acid	Others	1.21	3.82	1.93	up
pme3965	2-(dimethylamino)guanosine	Nucleotide and derivates	1.13	3.80	1.93	up
pme1814	2,5-dihydroxybenzoic acid	Organic acids and derivatives	1.18	3.36	1.75	up
pme2478	Protocatechuic aldehyde	Polyphenol	1.13	3.33	1.74	up
pme3198	2,4-Dihydroxybenzoic acid	Organic acids and derivatives	1.17	3.32	1.73	up
pme1824	Protocatechuic acid	Polyphenol	1.17	3.31	1.73	up
pme2706	2,3-Dihydroxybenzoic acid	Organic acids and derivatives	1.17	3.31	1.73	up
pme2761	4-Hydroxy-2-oxoglutaric acid	Organic acids and derivatives	1.19	2.87	1.52	up
pme2237	Dulcitol	Alcohols	1.16	2.69	1.43	up
pme0499	D-Sorbitol	Alcohols	1.14	2.68	1.42	up
pmb0889	Punicic acid	Lipids	1.15	2.64	1.40	up
pmf0280	D-Galacturonic acid	Organic acids and derivatives	1.20	2.50	1.32	up
pmb0758	Anisic acid O-feruloyl hexoside	Organic acids and derivatives	1.02	2.44	1.29	up
pmb2786	9-HOTrE	Lipids	1.09	2.29	1.19	up
pmb2981	Apigenin C-hexosyl-O-rutinoside	Flavone	1.06	2.27	1.19	up
pmf0265	5,7-Dihydroxychromone	Flavonoid	1.09	0.49	-1.03	down
pme0309	Methyl gallate	Organic acids and derivatives	1.07	0.47	-1.07	down
pme0279	2,6-Diaminooimelic acid	Amino acid and derivatives	1.15	0.47	-1.08	down
pme0422	3-Hydroxy-4-methoxycinnamic acid	Phenylpropanoids	1.00	0.46	-1.13	down
pmf0398	Arachidic acid	Lipids	1.19	0.45	-1.14	down
pme0239	2–furanoic acid	Organic acids and derivatives	1.09	0.42	-1.24	down
pme0038	Cytosine	Nucleotide and derivates	1.12	0.42	-1.24	down
pma2987	Histidinol	Alcohols	1.18	0.42	-1.25	down
pme1730	D-Erythronolactone	Organic acids and derivatives	1.20	0.41	-1.29	down
pme3419	Esculetin (6,7-dihydroxycoumarin	Phenylpropanoids	1.21	0.40	-1.33	down
pme1383	Pyridoxine	Vitamins and derivatives	1.02	0.39	-1.35	down
pme2636	Enterodiol	Alcohols	1.21	0.39	-1.37	down
pmb0882	LysoPC 18:1	Lipids	1.11	0.37	-1.43	down
pme2617	Methionine sulfoxide	Amino acid and derivatives	1.23	0.36	-1.46	down
pme0363	Chrysoeriol	Flavone	1.10	0.36	-1.47	down
pmb1650	Octadeca-11E,13E, 15Z-trienoic acid	Lipids	1.21	0.36	-1.48	down
pme1820	p–Hydroxyphenyl acetic acid	Organic acids and derivatives	1.10	0.35	-1.50	down
pmb0964	iP7G	Nucleotide and derivates	1.20	0.35	-1.53	down
pme3461	Homoeriodictyol	Flavanone	1.15	0.34	-1.55	down
pme2529	1,5-Anhydro-D-glucitol	Alcohols	1.15	0.34	-1.55	down
pmb2857	L-Glutamic acid O-glucoside	Amino acid and derivatives	1.19	0.34	-1.57	down
pme2380	A-Ketoglutaric acid	Organic acids and derivatives	1.21	0.32	-1.62	down
pmb3894	Di-O-methylquercetin	Flavonol	1.16	0.32	-1.63	down
pme2923	Acetoxyacetic acid	Organic acids and derivatives	1.05	0.32	-1.65	down
pme3292	Prunetin	Isoflavone	1.04	0.32	-1.66	down
pmd0160	LysoPE 16:0 (2n isomer)	Lipids	1.16	0.31	-1.67	down
pmb3117	LysoPE 16:0	Lipids	1.15	0.31	-1.70	down
pmd0017	Spermine	Phenolamides	1.21	0.30	-1.71	down
pme0379	Apigenin	Flavone	1.20	0.30	-1.72	down

pmb2855	L-Glutamine O-hexside	Amino acid and derivatives	1.22	0.30	-1.74	down
pme0496	Nicotinic acid	Vitamins and derivatives	1.14	0.29	-1.80	down
pme2601	3-Hydroxypropanoic acid	Organic acids and derivatives	1.01	0.28	-1.83	down
pmf0541	Shikonin	Quinones	1.19	0.28	-1.84	down
pme1021	D-(+)-Glucono-1,5-lactone	Carbohydrates	1.18	0.27	-1.87	down
pme0137	Nα-Acetyl-L-glutamine	Amino acid and derivatives	1.19	0.27	-1.89	down
pmb2654	Anthranilate O-hexosyl-O-hexoside	Organic acids and derivatives	1.05	0.27	-1.91	down
pme3250	Biochanin A	Isoflavone	1.06	0.26	-1.92	down
pmb0488	Spermidine	Phenolamides	1.15	0.26	-1.93	down
pme0486	Methylmalonic acid	Organic acids and derivatives	1.18	0.25	-2.00	down
pme3096	Aminomalonic acid	Organic acids and derivatives	1.18	0.25	-2.01	down
pme1830	Succinic acid	Organic acids and derivatives	1.18	0.25	-2.01	down
pme0291	Dl-2-Aminooctanoic acid	Organic acids and derivatives	1.21	0.24	-2.05	down
pme2949	Hesperetin 7-rutinoside (Hesperidin)	Flavanone	1.09	0.24	-2.07	down
pme1599	7-O-Methyleriodictyol	Flavanone	1.07	0.23	-2.09	down
pme2167	Orotic acid	Vitamins and derivatives	1.13	0.23	-2.12	down
pmf0274	Herbacetin	Flavonoid	1.17	0.23	-2.13	down
pme2596	4-Pyridoxic acid	Alkaloids	1.17	0.22	-2.19	down
pmb2826	Citramalate	Organic acids and derivatives	1.03	0.21	-2.22	down
pme2019	DL-Arabinose	Carbohydrates	1.14	0.21	-2.22	down
pmf0578	Citric acid monohydrate	Organic acids and derivatives	1.01	0.21	-2.24	down
pme0321	Kaempferol 7-O-rhamnoside	Flavonol	1.21	0.21	-2.25	down
pme0387	Homovanillic acid	Phenylpropanoids	1.18	0.21	-2.26	down
pmb2922	Uridine 5'-diphospho-D-glucose	Nucleotide and derivates	1.20	0.20	-2.29	down
pme3300	Tricetin	Flavone	1.15	0.20	-2.29	down
pmc0304	Succinyladenosine	Nucleotide and derivates	1.08	0.20	-2.33	down
pme0413	Vanillin	Organic acids and derivatives	1.21	0.20	-2.33	down
pme2009	L-(+)-Tartaric acid	Organic acids and derivatives	1.18	0.20	-2.34	down
pme1944	D-Mannitol	Alcohols	1.20	0.19	-2.36	down
pme1605	Kaempferol 3-O-robinobioside	Flavonol	1.18	0.19	-2.38	down
pme3336	N6-Succinyl Adenosine	Nucleotide and derivates	1.11	0.19	-2.40	down
pme0369	Kaempferol 3-O-rutinoside	Flavonol	1.15	0.19	-2.40	down
pme3393	Fustin	Flavonol	1.20	0.19	-2.43	down
pme3188	Uridine 5'-monophosphate	Nucleotide and derivates	1.19	0.17	-2.52	down
pme2241	Citraconic acid	Organic acids and derivatives	1.14	0.17	-2.57	down
pmf0059	UDP-α-D-glucose	Nucleotide and derivates	1.21	0.17	-2.59	down
pme1097	Adenine	Nucleotide and derivates	1.22	0.16	-2.60	down
pme2036	Quinic acid	Organic acids and derivatives	1.14	0.14	-2.80	down
pme1539	sorhamnetin 3-O-neohesperidosid	Flavonol	1.16	0.14	-2.80	down
pme0207	3-Hydroxybutyrate	Organic acids and derivatives	1.19	0.14	-2.81	down
pmf0118	Uridine 5'-diphosphoglucose disodium salt	Nucleotide and derivates	1.22	0.14	-2.82	down
pmf0179	Narcissoside	Flavonoid	1.11	0.14	-2.83	down
pmf0464	Farrerol	Flavonoid	1.09	0.14	-2.84	down
pme0183	2-Hydroxy-6-aminopurine	Nucleotide and derivates	1.20	0.14	-2.89	down
pmf0425	D-tartaric acid	Organic acids and derivatives	1.21	0.13	-2.90	down

pme1109	Guanine	Nucleotide and derivates	1.20	0.13	-2.92	down
pmf0282	Melibiose	Carbohydrates	1.15	0.13	-2.97	down
pme3719	D-Xylonic acid	Organic acids and derivatives	1.21	0.13	-2.98	down
pmc0960	LysoPC 20:4	Lipids	1.21	0.12	-3.01	down
pmf0175	2-Decanol	Alcohols	1.22	0.12	-3.02	down
pmf0174	1-Decanol	Alcohols	1.22	0.12	-3.03	down
pme0519	D-(+)-Sucrose	Carbohydrates	1.15	0.12	-3.05	down
pmb0859	LysoPC 18:1 (2n isomer)	Lipids	1.23	0.12	-3.08	down
pme3443	Sinapinaldehyde	Phenylpropanoids	1.20	0.12	-3.09	down
pmf0032	Galactinol	Carbohydrates	1.16	0.12	-3.11	down
pmb0962	Lysine butyrate	Amino acid and derivatives	1.04	0.11	-3.16	down
pme2050	Citric acid	Organic acids and derivatives	1.10	0.11	-3.25	down
pmb1912	10-Formyl-THF	Organic acids and derivatives	1.20	0.10	-3.37	down
pme2169	Fumaric acid	Organic acids and derivatives	1.20	0.09	-3.48	down
pme0033	Hypoxanthine	Nucleotide and derivates	1.22	0.09	-3.51	down
pme0275	4-Oxopentanoate	Organic acids and derivatives	1.19	0.07	-3.82	down
pme3313	D-Fructose 6-phosphate	Carbohydrates	1.14	0.07	-3.93	down
pmd0136	LysoPC 18:0	Lipids	1.22	0.06	-4.14	down
pme0534	Gluconic acid	Others	1.20	0.05	-4.31	down
pmb1754	O-Phosphocholine	Alkaloids	1.13	0.04	-4.52	down
pme3160	D-Glucose 6-phosphate	Carbohydrates	1.20	0.03	-4.97	down
pmf0035	Glucose-1-phosphate	Carbohydrates	1.18	0.03	-5.01	down
pmb0981	Adenosine 5'-monophosphate	Nucleotide and derivates	1.21	0.03	-5.08	down
pme3282	Afzelechin (3,5,7,4'-Tetrahydroxyflavan)	Flavanone	1.01	0.03	-5.15	down
pme0271	Maleic acid	Organic acids and derivatives	1.05	0.03	-5.20	down
pmb0514	Adenosine 3'-monophosphate	Nucleotide and derivates	1.22	0.03	-5.31	down
pmf0220	D-Fructose 6-phosphate-disodium salt	Carbohydrates	1.19	0.02	-5.36	down
pme1175	Guanosine	Nucleotide and derivates	1.03	0.02	-5.45	down
pme2049	2-Hydroxybutanoic acid	Organic acids and derivatives	1.22	0.02	-5.49	down
pmb2657	Argininosuccinate	Organic acids and derivatives	1.14	0.02	-5.52	down
pme0004	L-Homocitrulline	Amino acid and derivatives	1.03	0.02	-5.58	down
pmb3264	Glutathione oxidized	Amino acid and derivatives	1.05	0.02	-5.62	down
pmb3081	Glucarate O-Phosphoric acid	Others	1.02	0.02	-5.64	down
pmb4344	Guanosine 5'-monophosphate	Nucleotide and derivates	1.06	0.02	-5.71	down
pme1063	Uridine	Nucleotide and derivates	1.19	0.02	-5.76	down
pmf0326	Apocynin	Phenylpropanoids	1.03	0.01	-6.18	down
pme0056	2,3-dimethylsuccinic acid	Amino acid and derivatives	1.23	0.01	-6.22	down
pme2033	L(-)-Malic acid	Organic acids and derivatives	1.22	0.01	-6.24	down
pme3309	2-Methylglutaric acid	Organic acids and derivatives	1.23	0.01	-6.27	down
pmb3088	Trehalose 6-phosphate	Carbohydrates	1.20	0.01	-6.41	down
pmb0854	LysoPC 18:3	Lipids	1.22	0.01	-6.50	down
pmb0532	Inosine 5'-monophosphate	Nucleotide and derivates	1.06	0.01	-6.52	down
pmb0855	LysoPC 16:0	Lipids	1.22	0.01	-6.59	down
pme3098	4-Methylvaleric acid	Organic acids and derivatives	1.09	0.01	-6.67	down
pmd0132	LysoPC 16:0 (2n isomer)	Lipids	1.22	0.01	-6.68	down

pmf0217	D-Glucose-6-phosphate disodium salt	Others	1.22	0.01	-6.82	down
pme2541	α-Hydroxyisobutyric acid	Organic acids and derivatives	1.07	0.01	-6.83	down
pmf0485	Panose	Carbohydrates	1.21	0.01	-6.95	down
pme0243	Glutaric acid	Organic acids and derivatives	1.22	0.01	-7.00	down
pme2129	(S)-(-)-2-Hydroxyisocaproic acid	Organic acids and derivatives	1.20	0.01	-7.01	down
pma6270	sn-Glycero-3-phosphocholine	Alkaloids	1.21	0.01	-7.03	down
pme0267	2-Methylsuccinic acid	Organic acids and derivatives	1.22	0.01	-7.20	down
pme0250	Azelaic acid	Organic acids and derivatives	1.21	0.01	-7.62	down
pmf0348	2,6-Dimethyl-7-octene-2,3,6-triol	Alcohols	1.22	0.00	-7.64	down
pme1977	Suberic acid	Organic acids and derivatives	1.22	0.00	-8.43	down
pme3235	Liquiritigenin	Flavanone	1.22	0.00	-10.98	down
pme2935	2-Oxovaleric acid	Organic acids and derivatives	1.23	0.00	-11.62	down
pmb2928	Gallic acid O-Hexoside	Organic acids and derivatives	1.23	0.00	-12.04	down
pmb0588	Luteolin 3',7-di-O-glucoside	Flavone	1.23	0.00	-12.42	down
pmf0098	trans-4-Hydroxycinnamic acid Methyl Ester	Phenylpropanoids	1.23	0.00	-12.76	down
pme2284	Pentadecafluorooctanoic acid	Organic acids and derivatives	1.23	0.00	-12.78	down
pme0173	N-Propionylglycine	Amino acid and derivatives	1.23	0.00	-13.08	down
pmf0513	Ruscogenin	Sterides	1.23	0.00	-13.35	down
pmd0023	Adenosine	Nucleotide and derivates	1.23	0.00	-13.61	down
pma2405	Feruloylcholine	Alkaloids	1.23	0.00	-13.71	down
pmb2228	LysoPC 19:0	Lipids	1.23	0.00	-13.73	down
pmb3079	N-Acetylglucosamine 1-phosphate	Others	1.22	0.00	-13.75	down
pme1299	Dl-3,4-Dihydroxymandelic acid	Organic acids and derivatives	1.23	0.00	-13.85	down
pme1711	3-Hydroxy-3-methyl butyric acid	Organic acids and derivatives	1.23	0.00	-13.87	down
pme2879	5-Hydroxymethyluracil	Nucleotide and derivates	1.23	0.00	-14.01	down
pme0180	1-Methylhistidine	Amino acid and derivatives	1.23	0.00	-14.10	down
pme0266	Sebacate	Organic acids and derivatives	1.23	0.00	-14.15	down
pma0294	Chrysoeriol 5-O-hexoside	Flavone	1.23	0.00	-14.29	down
pma6639	Isorhamnetin O-hexoside	Flavonol	1.23	0.00	-14.31	down
pmb0607	Chrysoeriol 7-O-hexoside	Flavone	1.23	0.00	-14.34	down
pmf0374	Isorhamnetin 3-O-glucoside	Flavonoid	1.23	0.00	-14.36	down
pme1322	Nα-Acetyl-L-arginine	Amino acid and derivatives	1.23	0.00	-14.38	down
pmb0595	Isorhamnetin 5-O-hexoside	Flavonol	1.23	0.00	-14.51	down
pme3154	(Rs)-Mevalonic acid	Organic acids and derivatives	1.23	0.00	-14.58	down
pme3174	Cytidine 5'-monophosphate (Cytidylic acid)	Nucleotide and derivates	1.22	0.00	-14.58	down
pmb0542	Cyanidin 3-O-malonylhexoside	Anthocyanins	1.23	0.00	-14.63	down
pme1294	Xanthosine	Nucleotide and derivates	1.23	0.00	-14.67	down
pmf0418	Curdione	Others	1.23	0.00	-15.28	down
pmb0696	8-C-hexosyl chrysoeriol O-hexoside	Flavone	1.23	0.00	-15.69	down
pme3263	2'-Hydroxydaidzein	Isoflavone	1.23	0.00	-15.93	down
pme3034	ethylmalonate	Organic acids and derivatives	1.23	0.00	-16.32	down
pme3197	Cyclic AMP	Nucleotide and derivates	1.22	0.00	-16.33	down
pme0391	4-Methylumbelliferone	Phenylpropanoids	1.23	0.00	-16.80	down
pme3827	3,4-Dihydroxy-DL-phenylalanine	Amino acid and derivatives	1.23	0.00	-16.93	down

pmb2871	2,5-dihydroxy benzoic acid O-hexside	Organic acids and derivatives	1.23	0.00	-17.28	down
pmb2831	Protocatechuic acid O-glucoside	Polyphenol	1.22	0.00	-17.62	down
pmb2406	LysoPC 17:0	Lipids	1.23	0.00	-17.82	down
pmf0173	1,2-Decanediol	Alcohols	1.23	0.00	-18.44	down
